# HOXB3 drives WNT-activation associated progression in castration-resistant prostate cancer

**DOI:** 10.1038/s41419-023-05742-y

**Published:** 2023-03-27

**Authors:** Shimiao Zhu, Zhao Yang, Zheng Zhang, Hongli Zhang, Songyang Li, Tao Wu, Xuanrong Chen, Jianing Guo, Aixiang Wang, Hao Tian, Jianpeng Yu, Changwen Zhang, Lei Su, Zhiqun Shang, Changyi Quan, Yuanjie Niu

**Affiliations:** 1 Department of Urology, Tianjin Institute of Urology, The Second Hospital of Tianjin Meidical University, Tianjin, 300211 China; 2 Department of Urology, Shenzhen Hospital, Southern Medical University, Shenzhen, China; 3 Department of Pathology, The Second Hospital of Tianjin Medical University, Tianjin, China; 4 Institute of Urology, Peking University; National Urological Cancer Center, Beijing, China; 5 Department of Urology, People’s Hospital of Rizhao, Rizhao, Shandong China

**Keywords:** Prostate cancer, Translational research, Prostate cancer

## Abstract

Enabled resistance or innate insensitiveness to antiandrogen are lethal for castration-resistant prostate cancer (CRPC). Unfortunately, there seems to be little can be done to overcome the antiandrogen resistance because of the largely unknown mechanisms. In prospective cohort study, we found that HOXB3 protein level was an independent risk factor of PSA progression and death in patients with metastatic CRPC. In vivo, upregulated HOXB3 contributed to CRPC xenografts progression and abiraterone resistance. To uncover the mechanism of HOXB3 driving tumor progression, we performed RNA-sequencing in HOXB3 negative (HOXB3-) and HOXB3 high (HOXB3 + ) staining CRPC tumors and determined that HOXB3 activation was associated with the expression of WNT3A and enriched WNT pathway genes. Furthermore, extra WNT3A and APC deficiency led HOXB3 to be isolated from destruction-complex, translocated to nuclei, and then transcriptionally regulated multiple WNT pathway genes. What’s more, we also observed that the suppression of HOXB3 could reduce cell proliferation in APC-downregulated CRPC cells and sensitize APC-deficient CRPC xenografts to abiraterone again. Together, our data indicated that HOXB3 served as a downstream transcription factor of WNT pathway and defined a subgroup of CRPC resistant to antiandrogen which would benefit from HOXB3-targeted therapy.

## Introduction

The progression of castration-resistant prostate cancer (CRPC) is intrinsically dependent on androgens and androgen receptor (A/AR) signaling, as a result, antiandrogen therapy is still the backbone of systemic treatment of this disease [1]. In addition to A/AR-addicted manners, many other mechanisms are involved in the development of CRPC. One of the relevant A/AR-independent mechanism is activation of WNT pathway [2], which reminds us of that non-A/AR targeted therapies are needed, especially for these patients received antiandrogen therapy in the earlier disease stages.

The homeobox (HOX) proteins, containing a highly conserved homeodomain with a conserved helix-turn-helix motif that binds to the DNA, have long been known as crucial factors of gene transcription during development [3]. Recently, increased evidence showed HOX proteins played important roles in cancer initiation and progression [4, 5]. As one of the typical HOX protein, HOXB3 has been proven to be associated with tumor progression in hepatocellular carcinoma and breast cancer [6, 7]. In prostate cancer (PCA), our previous study observed that HOXB3 could promote tumor recurrence in hormone-naïve PCA partly through transactivating CDCA3 expression [8]. However, we still don’t know what role it plays in progression and resistance to antiandrogens in CRPC. Furthermore, the mechanisms of regulating HOXB3 protein activity and pathways/genes regulated by HOXB3 are also not being revealed.

As a significant regulator of growth, development, and tissue homeostasis, the WNT signaling pathway is involved in many physiological and pathological processes throughout the life [9]. WNT signaling can be activated by WNT3A, a classic canonical WNT ligand, through eliciting β-catenin/TCF [2]. When ligand is absent, WNT pathway can also be activated by disrupting ‘destruction complex’ that composed by adenomatous polyposis coli (APC), AXIN1, beta-transducing repeat-containing protein (β-TrCP), and phosphorylating kinases, such as glycogen synthase kinase 3 (GSK3), KappaB kinase β (IKKβ), and/or casein kinase Iα (CKIα) [9, 10]. The β-catenin/TCF target genes account for majority biofunctions of WNT-signaling pathway, however, the highly temporal and spatial expression of these genes is not enough to explain the complexity and universality of WNT biology [9, 11, 12].

In this study, we observed that HOXB3 protein activity is associated with the resistance to first line abiraterone in a prospective cohort of mCRPC patients. Additionally, the stabilization and activation of HOXB3 protein could be induced by WNT-pathway activation. These findings are consistent with the result of association between WNT-pathway mutations and abiraterone resistance in mCRPC [13]. Together, our data indicate that HOXB3 protein is demonstrated to be alternatively transcriptive effector of WNT signaling that is responsible for tumor progression in WNT-activated CRPC and may serve as a potential therapeutic target for the subgroup of patients.

## Methods

### Patients

Patients were collected from an observational, prospective, multi-center study of mCRPC patients, with a starting of abiraterone as first line novel hormonal therapy (NHT) (No. NCT03176381). Enrollment took place between May 2017 and May 2021 in 6 Chinese urological centers. The study was conducted according to the Declaration of Helsinki and principles of good clinical practice, with the approvement of the ethics committees of Tianjin Medical University Second Hospital. The main selection criteria were as follows: (1) Men have given consent form aged ≥18 years; (2) Patients with a confirmed diagnosis of mCRPC according to EAU 2017 guideline; (3) Patients with biopsy-acquired CRPC tissues for immunohistochemistry (IHC) staining and RNA-sequencing (RNA-seq); (4) Participants with an Eastern Cooperative Oncology Group (ECOG) 23 performance status score of 2 or less; (5) Patients with hematologic values that met predefined criteria, including an albumin ≥3.0 g/dL. The exclusion criteria were as follows: (1) Participants are allergic to contrast agent; (2) Patients were excluded if they planned to receive additional concurrent anticancer medicine; (3) Patients have received other NHT before abiraterone; (4) Patients were also excluded if they had coexisting malignant, serious nonmalignant disease, active viral hepatitis, chronic liver disease, abnormal aminotransferase levels (≥2.5 times the upper level of normal range for patients without known liver metastasis; ≥5 times the upper level of normal range for patients with known liver metastasis), clinically significant heart disease, or dysfunction of pituitary or adrenal.

Baseline characteristics of all patients were collected, including demographic, pathologic and clinical information. The primary endpoint was time to prostate specific antigen (PSA) progression that defined as the time from the starting of abiraterone therapy to the second 50% increasement in PSA above the nadir at a minimum of 2 ng/mL. Secondary endpoints were overall survival (OS) that defined as the time from starting abiraterone therapy to death from any cause.

### Tumor specimens, immunohistochemistry of tissues, scoring

Formalin-fixed paraffin-embedded (FFPE) or flash-frozen tumor tissues were collected from secondary biopsy on metastases. Prostate biopsy was permitted when tissue from metastasis wasn’t available. The detailed principle for metastatic biopsy was descripted in our previous work [14]. The FFPE tissues were used for IHC, fresh ones were exploited to RNA-seq. IHC staining and scoring were performed as previously described [15]. After deparaffinization, 3% H_2_O_2_-treatment, antigen retrieval and blockage in goat serum, slides were incubated with primary antibodies, labeled secondary antibodies and DAB (Zhong Shan gold bridge, Beijing). Stained tumor sections were scored independently by two pathologists (WA & GJ) blinding to patients’ clinical characteristics. A 0 to 6 scale was used for nuclear-staining score according to intensity and quantity score, with 0/1 for negative/low staining, 2 or 3 for intermediate, and 4 or 6 for high nuclear staining. Nuclear staining intensity and quantity were reviewed in five random medium magnifications. Quantity was evaluated based on percentage of stained nuclear: 0% = 0 points; <25% = 1 point; ≥25% = 2 points. Intensity was graded based on option density: negative = 0 points; light brown coloration = 1 point; brown = 2 points; dark brown with unstained background = 3 points. Discrepant results were re-assessed and discussed until a consensus was got.

### RNA-sequencing and analysis

Samples with negative HOXB3 staining (HOXB3-, *n* = 10) or score 2 staining (HOXB3 + , *n* = 10) were chosen to undergo RNA-seq and Analysis as described previously [16]. Briefly, nucleic acids were extracted from the tissues with highest tumor content as determined by pathology review. Total RNA was extracted from tumors using RNeasy Plus Mini Kit (QIAGEN). RNA quality was verified by Agilent Bioanalyzer using NanoDrop 8000 (Agilent Technologies). Only samples with minimum RIN (RNA integrity number) score of 7 were permitted to sequence. Transcriptome libraries were prepared from 500–1000 ng riboRNA-depleted RNA using TruSeq Stranded RNA/Ribo-Zero Sample Preparation Kit following the Illumina protocol.

Fragments with 250–300 bp were selected on FlashPAGE denaturing PAGE-fractionator (Life Technologies) before ethanol precipitation overnight. After ligation, reverse-transcription, and RNase H treatment, samples were measured in Illumina HiSeq 4000 platform. Quality control was performed using FastQC (RRID:SCR_014583) and RSeQC (RRID:SCR_005275), then pair-end raw reads were mapped onto the human reference genome (GRch37.65) using the TopHat algorithm (v2.0.9, RRID:SCR_013035). The gene-level transcriptions were shown as log2(x + 1) transformed FPKM [17]. Further analyses were performed using the R (v2.15.0). To determine the difference of the genes in two groups, we used DEGseq package (RRID:SCR_008480) to render the absolute value of the log_2_(ratio) of HOXB3 + *vs*. HOXB3- wasn’t less than 2 with *p* < 0.05.

### Primary antibodies, cell culture, and reagents

Antibodies for WNT3A (#2391), β-TrCP (#4394), AXIN1 (#2087), APC (#2504), ERK1/2 (#9102), AKT (#4691), MEK1/2 (#9122), phosphor-ERK1/2 (#9101), phosphor-AKT (#4060), phosphor-MEK1/2 (#9154) and IKKβ (#8943) were purchased from Cell Signaling; antibody for β-catenin (610153) was from BD Biosciences; antibodies for CXCL14 (ab264467), CXCR2 (ab89254), MMP2 (ab92536), MMP10 (ab261733), FGF4 (ab65974) and NCAM1 (ab220360) were from Abcam; antibody for HOXB3 (PA5-103890) was from Invitrogen, antibodies for CKIα (H-7, sc-74582) and GSK3 (0011-A, sc-7291) were from Santa Cruz; antibody for β-actin (A5441) was from Sigma. Recombinant protein WNT3A was from R&D Systems (100 ng/ml). IWR-1, DMSO and other chemicals were purchased from Sigma-Aldrich unless otherwise stated.

LNCaP (RRID:CVCL_0395), C4-2 (RRID:CVCL_4782) and HEK293 (RRID:CVCL_0045) cells were obtained from the American Type Culture Collection. C4-2 were cultured in RPMI 1640 (Gibco) supplemented with 10% charcoal-stripped fetal bovine serum (CSS-FBS) (BI) and 100 μg/ml penicillin-streptomycin-glutamine (Invitrogen). HEK293 was cultured in DMEM (Gibco) supplemented with 10% FBS. LNCaP-AI were established following long-term culture of the parental LNCaP cells under androgen-deprived conditions (RPMI 1640 medium supplemented with 10% CSS-FBS). C4-2AR (Abiraterone resistant C4-2) were maintained in RPMI 1640 medium with 10% CSS-FBS contain 10 μM abiraterone actate (APExBIO). All of these cells were incubated under a humidified atmosphere at 37 °C with 5% CO2.

### Plasmids construction, shRNA knockdown, siRNA interference and luciferase assays

The full-length HOXB3 cDNA were reverse transcriptase-polymerase chain reaction using total RNA from LNCaP-AI cell line. The primer sequences for HOXB3 were 5′-GAATTCATGCAGAAAGCCACCTACTAC-3′ (forward) and 5′-CTCGAGTCACAGGTGTGTTAATTTG-3′ (reverse). The cDNA products were cloned into EcoR I and Xho I sites of the mammalian expression vector pcDNA3.1 (Invitrogen) (pcDNA3.1-HOXB3). The HOXB3 shRNA and APC shRNA plasmids were purchased from GeneCopoeia (HOXB3: HSH100328-LVRH1GP; APC: HSH100361-LVRU6MH).

The siRNA-insensitive Flag-HOXB3 and Myc-AXIN1 were both cloned in pcDNA3.1 using standard PCR procedures with A3906 and E2160 cDNAs (Gene Copoeia) as templates. Point mutations were induced by PCR using the QuikChange Site-directed Mutagenesis following Agilent protocols. Three deletion constructs were made from above Flag-HOXB3 by PCR with specific primers.

After infection/transfection, stable clones were selected with 1 mg/ml puromycin or 600 U/ml hygromycin B. Knockdown (>75%) was confirmed by qRT-PCR or western blot. siRNAs targeting HOXB3, β-catenin, β-TrCP, AXIN1, APC, CKIα, GSK3 and IKKβ and negative controls were purchased from Santa Cruz. Transfections were conducted using Lipofectamine RNAiMAX (Invitrogen).

Luciferase assay was performed with the established CDCA3 reporter [8] or β-catenin/TCF-responsive reporter TOP-FLASH [18]. Promoters’ region representative WNT-regulated genes were amplified from genomic DNA of LNCaP-AI cells and the fragment was cloned into the Bgl II and Kpn I restriction sites in pGL3-basic vector (Promega, luciferase reporter plasmids). All constructs were verified by qRT-PCR. Luciferase reporter plasmids and CMV-β-gal were transfected together for transfection efficiency with CPRG (Roche) colorimetic assay.

### Quantitative real-time PCR

Total RNA was extracted from tissues and cells using TRIzol reagent (Invitrogen). qRT-PCR was performed on BioRad iCycler iQ (Bio-Rad Laboratories, RRID:SCR_008426) using SYBR Green Supermix (Bio-Rad Laboratories). The primer sets were used at a concentration of 0.2 µM. All PCR assays were performed duplicately with no less than three independent samples. Expression levels were normalized to GAPDH. All primers were designed using primer 3 (https://bioinfo.ut.ee/primer3/) and synthesized by Sangon Biotech (Shanghai, China). Primers for human samples were listed in Table S5.

### Immunoblotting and immunoprecipitation (CO-IP)

Protein was isolated from tissues and cells using RIPA lysis buffer. Protein lysates were stratified in 8%–12% Bis-Tris Gel, transferred to PVDF membranes and probed with HRP-labeled secondary antibodies (GE Healthcare). Protein expressions were visualized with ECL reagent (Thermo Scientific).

For protein-protein interaction studies, cells were incubated with lysis buffer (pH 7.4, 5 mM HEPES, 150 mM NaCl, 5 mM PMSF, 1 mM EDTA, 1% Triton X-100, 10 mM glycerol and 2 mM Na3VO4) at 4 °C for 30 min and centrifuged at 13,000 rpm for 15 mins. After the concentration was measured by BCA Assay, 500 μg of protein incubated with 2–3 μg primary antibodies under moderate swing overnight at 4 °C, and then with 50 μl protein A Sepharose beads for an additional 2 h. After being collected by magnet, immunocomplexes were washed 3 times with cold lysis buffer, resuspended in SDS sample buffer, boiled for 5 mins, and subsequently subjected to SDS-PAGE and Western blot analysis [19]. A replication of the experiment was performed at the last.

### GST pull-down

For GST-pull down, HOXB3 or its truncated mutants were cloned into the pGEX4T1 vector containing N-terminal GST (RRID: Addgene_121101). All constructs were confirmed by sequencing. After cloning, the fusion proteins were purified from BL-21 following the protocol descripted [20]. Beads with purified GST-HOXB3 was incubated with 35S-methionine-labeled in vitro-translated AXIN1 for 3 h in Binding Buffer (PH 7.9 HEPES, 5 mM EDTA, 0.4 M KCl, 0.4% NP40, 1 mM DTT and 10% glycerol with protease inhibitors). The copurified proteins were washed 4 times with binding buffer and subjected to SDS-PAGE and Western blot analysis with anti-GST antibody (B-14, sc-138).

### Immunofluorescence staining

After deparaffinization, antigen retrieval and blockage in 10% goat serum, slides were incubated with primary antibodies and secondary antibodies. Secondary antibodies were Donkey anti-rabbit coupled to Alexa-488 (Invitrogen, A32766). Cell nuclei were visualized with DAPI (Sigma). The visualization was acquired achieving with Olympus FV1000D confocal microscope.

### Chromatin immunoprecipitaion (ChIP)

CHIP assays were was carried out using modified CHIP Assaykit (Upstate) mainly according to previous description [8]. In brief, LNCaP-AI cells were quenched with 0.125 M glycine for 5 min after crosslink with 1% formaldehyde. Cells were then washed with ice-cold PBS and lysed in lysis buffer. Harvested lysates were centrifuged with a rotating speed of 2300 × g at 4 °C for 5 min. The pelleted chromatin DNAs were digested in ChIP MNase buffer, followed by a final concentration of 10 mM EDTA to stop the reaction. The harvested samples were sonicated to shear the DNA to a range of 500 bp to 1000 bp. After removing debris by the second centrifugation (16,000 × g for 10 min at 4 °C), acquired supernatants were diluted to 1:5 in CHIP dilution buffer (20 mM Tris-HCl pH 8.0, 150 mM NaCl, 1 mM EDTA, and 1% Triton-X-100). About 5% of the chromatin extract was set aside for input.

The majority chromatin extracts were immunoprecipitated at 4 °C overnight with 2 μg anti-HOXB3 or control rabbit IgG that were pre-treated with 50 μl of anti-rabbit IgG magnetic beads. The processed beads were sequentially washed with low salt wash buffer (20 mM Tris-HCl pH 8.0, 150 mM NaCl, 2 mM EDTA, 1% Triton-X-100), high salt wash buffer (20 mM Tris-HCl pH 8.0, 500 mM NaCl, 2 mM EDTA, 1% Triton-X-100), LiCl wash buffer (250 mM LiCl, 20 mM Tris-HCl pH 8.0, 1 mM EDTA, 1% NP-40, 1% sodium deoxycholate), and TE buffer (100 mM Tris-HCl pH 8.0, 10 mM EDTA). Then, beads were incubated in elution buffer (50 mM Tris-HCl pH 8.0, 10 mM EDTA, 1% SDS) for 30 min at 65 °C. After reversal of crosslinking overnight at 65 °C, samples were digested with RNase A (0.2 mg/ml) and proteinase K (0.2 mg/ml). Finally, DNA was purified by using PCR Purification Kit (Qiagen) followed by qRT-PCR according to the manufacture’s instruction

### Generation of LAPCi-HOXB3^Tet^ and C4-2APCi-HOXB3^Tet^ cells

LNCaP-AI-shAPC and C4-2-shAPC cells were infected with lentivirus to integrate a doxycycline inducible HOXB3 shRNA (TetIIP-GFP-shHOXB3(MIR30)-Ubi-TetR-IRES-Puromycin) using the GV553-Tet-On backbone (Genechem, Shanghai, China). Selection of stably transfected cells was achieved using 2 ug/mL of puromycin followed by clone-selection in 96-well plates. Selected LNCaP-AI-shAPC with effectiveness of HOXB3 suppression was called LAPCi-HOXB3^Tet^ for short, while C4-2-shAPC with HOXB3 knockdown was named C4-2APCi-HOXB3^Tet^ for short. Control cells were obtained by using lentivirus integrated with doxycycline inducible scramble shRNA.

### Murine prostate tumor xenograft model

All animal experiments were performed in compliance with University Committee on Use and Care of Animals at Tianjin Medical University conforming to regulatory standards and were approved by the institutional ethics committee. Seven-week-old male immunocompromised (nonobese diabetic [NOD]-severe combined immunodeficiency [SCID]) mice were procured from a breeding colony at Chinese Academy of Sciences maintained at animal laboratory of Tianjin Institute of Urology. All of these NOD-SCID mice were castrated through cutting off their testes before xenograft. A week later, mice were subcutaneously implanted 5 × 10^6^ LA-Control, 5 × 10^6^ LA-HOXB3, 5 × 10^6^ LAPCi-HOXB3^Tet^ or 5 × 10^6^ C4-2APCi-HOXB3^Tet^ cells suspended in 50 μl PBS with 1:1 Matrigel (BD Biosciences). Until the tumors grew and reached a size of approximately 100 mm^3^, mice were randomized to receive drinking water with 0.5% sucrose supplemented Doxycycline (0.5 mg/mL, Sigma) or not [21], with or without abiraterone acetate (200 mg/kg, p.o.). At day 28, the animals were sacrificed, then the tumors were removed and measured by investigators in a blind manner, with no less than 6 mice in each group.

### Statistical analysis

The survival data were visualized by Kaplan-Meier curves. Cox proportional hazard models were used in the univariate and multivariate analyses of time to PSA progression and OS. The results of continuous variable were displayed as mean ± SD. Paired comparisons between two groups were performed using Student’s t test. ANOVA was used to compare multiple groups. R-4.0.0. was used to carry out those analyses. Data visualizations were realized by GraphPad-prism 8 and R package ggplot2 (RRID:SCR_014601). Statistical tests were two sided. *P* < 0.05 was considered significant.

### Experiment replicates

RT-PCR, luciferase report, western blots, CO-IP, and GST pull-down experiments were repeated three times. Three replicates were launched for RT-PCR each time. MTT and invasion assays were done three times, with six replicates for each group.

## Results

### HOXB3 is associated with the resistance to abiraterone and poor outcome in mCRPC patients

**Fig. 1:**
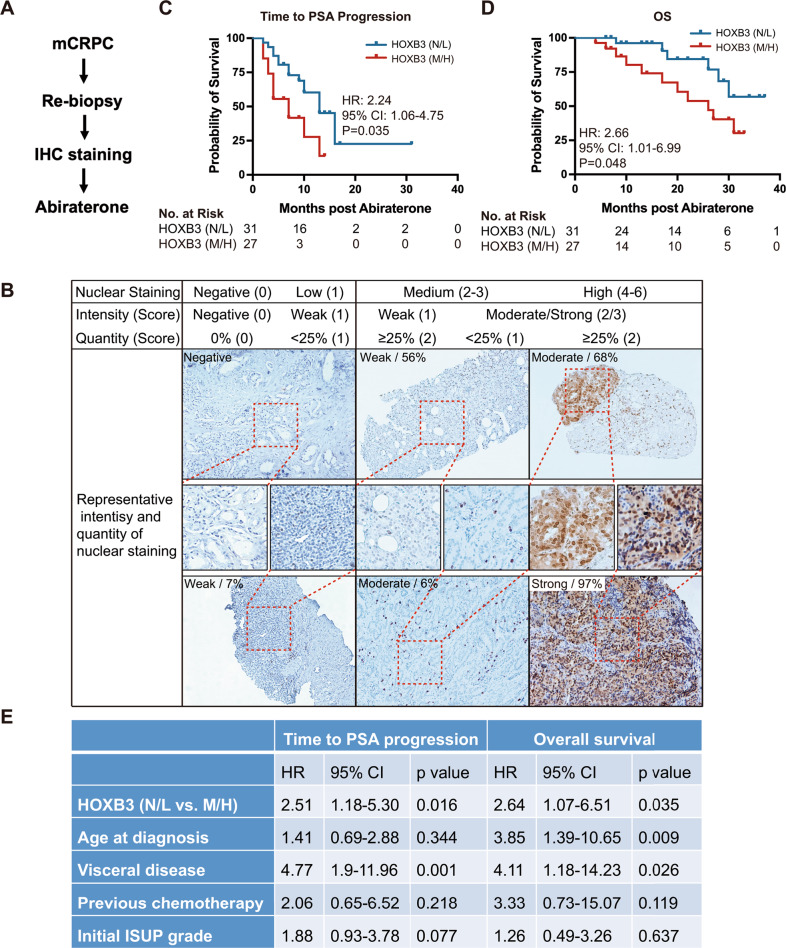
HOXB3 expression is associated with resistance to abiraterone and poor outcome in mCRPC patients. **A** Flow chart of patients participating in this prospective cohort study. **B** Representative staining of HOXB3 in nuclear. Staining levels were classified as Negative or Low (N/L), Medium and High. **C**, **D** Kaplan–Meier curves of (**C**) time to PSA progression and (**D**) overall survival (OS) after abiraterone therapy in CRPC patients, by HOXB3 expression. **E** Multivariable logistic regression analyses of time to PSA progression and OS. IHC immunohistochemistry, CI confidence interval, HR hazard ratio, ISUP International Society of Urological Pathology Consensus Conference on Prostate Cancer Grading.

Our previous findings in hormone-naive local PCA indicated that HOXB3 mRNA expression correlated with tumor grade,stage and tumor recurrence after radical prostatectomy [8]. Its predictive value in advanced disease receiving systemic therapy is still largely unknown. Considering that HOXB3 may be upregulated posttranscriptionally without a change in its mRNA level, we provided an assessment of its protein level in nucleus by immunohistochemistry (IHC) in biopsy samples from a prospective cohort of mCRPC patients who received abiraterone as first line NHT (Fig. 1A, NCT03176381). HOXB3 nuclear protein level was scored in a blinded manner based on staining intensity and quantity of nuclear HOXB3 as predefined. The representatives of HOXB3 staining intensity and quantity were shown in Fig. 1B. A total of 58 patients were recruited, with sufficient pathological tissue was used to IHC and RNA-seq, as well as a no less than one year of clinical follow-up.

Of these patients, 46.6% (27 patients) harbored potentially medium/high nuclear staining of HOXB3 (M/H group), including 17.2% (*n* = 10) with high and 29.3% (*n* = 17) with medium level of nuclear staining (Table S1). The remaining 53.4% patients (*n* = 21) had negative/low staining of HOXB3 (N/L group), including 17.2% (*n* = 10) with negative HOXB3 staining. Baseline clinical, demographic, and pathologic characteristics were listed in Table S1 according to nuclear HOXB3 staining. In this data set, significantly more visceral diseases could be seen in HOXB3 M/H group patients (Table S1). We further evaluate whether HOXB3 nuclear staining is associated with disease progression and survival in abiraterone-treated mCRPC patients. The median time to PSA progression and OS of the whole cohort was 10 months (7 months in M/H group; 13 months in N/L group) and 31 months (26 months in M/H group; undefined in N/L group), respectively. We found markedly reduced time to PSA progression (HR 2.24, 95% CI 1.06–4.75, *p* = 0.035), as well as lower OS (HR 2.66, 95% CI 1.01–6.99, *p* = 0.048), in patients whose tumors had high or medium levels of nuclear HOXB3 staining (Fig. 1C, D). Moreover, in a multivariable model, HOXB3 nuclear staining as an independent factor was related to decreased time to PSA progression (HR 2.51, 95% CI 1.18–5.30, *p* = 0.016). Visceral disease was also independently connected to higher hazard of PSA progression (HR 4.77, 95% CI 1.90–11.96, *p* = 0.001). OS was also significantly shorter in patients with high or medium levels of nuclear HOXB3 staining in multivariable analysis (HR 2.64, 95% CI 1.07–6.51, *p* = 0.035). Other factors were also detected to be independently relevant to increased hazard of death in multivariable analysis, such as age and Visceral disease. The multivariable results for time to PSA progression and OS can be found in Fig. 1E.

### HOXB3 promotes proliferation and contributes to abiraterone resistance in vitro and in vivo

**Fig. 2:**
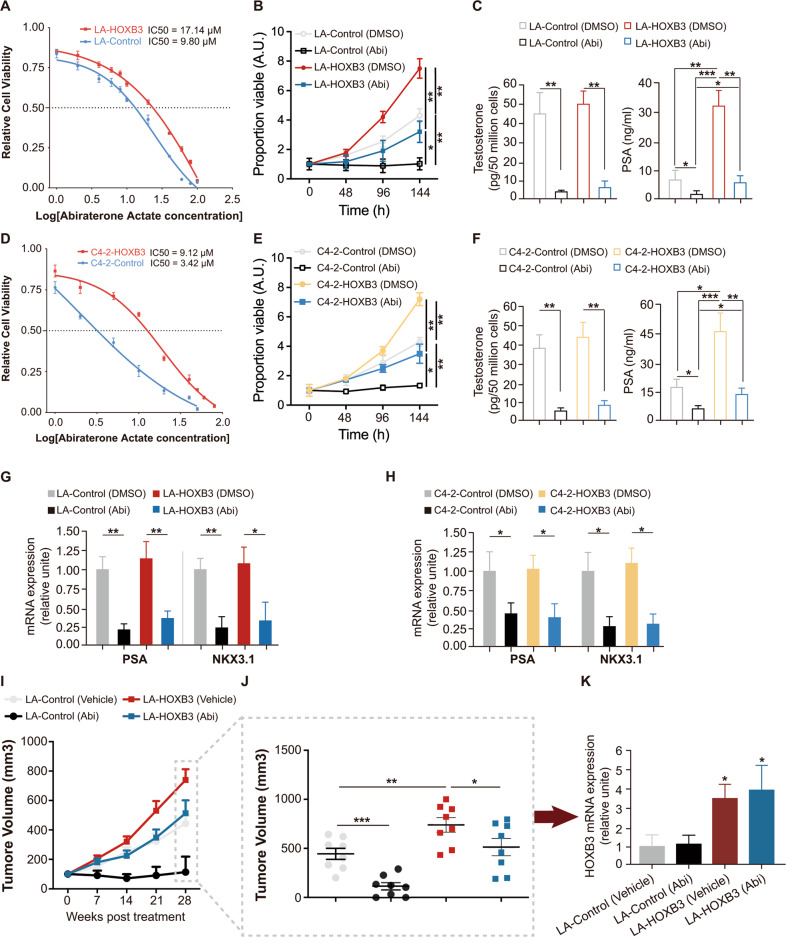
HOXB3 promotes CRPC resistance to abiraterone in vitro and in vivo, see also Fig. S1. **A**, **D** Cell growth in the presence of different concentrations of abiraterone actate was measured in LA-Control/LA-HOXB3 and C4-2-Control/C4-2-HOXB3. IC50 values are indicated by dotted lines. **B**, **E** MTT assays were performed in LA-Control/LA-HOXB3 or C4-2-Control/C4-2-HOXB3 cells after treated with DMSO or 10 μmol/L abiraterone (Abi) for indicated time. **C**, **F** LA-Control/LA-HOXB3 or C4-2-Control/C4-2-HOXB3 cells were treated with DMSO or 10 μmol/L Abi in charcoal stripped serum for 96 h. Fifty million cells were collected from each group followed by testosterone level examination by LC-MS, and the supernatants were subjected to PSA value test by ELISA. **G**, **H** qRT-PCR assays were performed in LA-Control/LA-HOXB3 or C4-2-Control/C4-2-HOXB3 cells after treated with DMSO or 10 μmol/L abiraterone (Abi) for 96 h. **I** LA-Control or LA-HOXB3 cells were injected subcutaneously in mice and grown until tumors reached a size of ~100 mm^3^. Xenografted mice were then randomly treated with vehicle control or abiraterone acetate (200 mg/Kg, p.o). Caliper measurements were taken weekly. *n* = 8 mice per group. **J** Scatter plots with bar showing individual tumor volumes at 28 days post-randomization in each group (*n* = 8 per group). **K** Bar graph showing HOXB3 stable suppression in indicated tumors at 28 days post-randomization. HOXB3 mRNA levels were measured by qRT-PCR. Error bars indicate mean ± SD. *p < 0.05, **p < 0.01, ***p < 0.001.

Our data in clinical cohorts established that M/H nuclear staining of HOXB3 is associated with tumor progression and poor outcome in abiraterone-treated mCRPC patients. Thus, we hypothesized that HOXB3 contributes to abiraterone resistance in CRPC. To corroborate this hypothesis, we stably overexpressed HOXB3 in LNCaP-AI and C4–2 cells which have low protein level HOXB3 and sensitive to abiraterone (Fig. S1A, B). LNCaP-AI cell was a kind of androgen independent LNCaP cell that induced by castration in vitro [19]. The dose-response curves were generated in LNCaP-AI and C4-2 by assessing cell viability and IC50 values were calculated. Indeed, IC50 value of abiraterone for LA-HOXB3 (IC50 value = 17.14 μM) was higher than that of LA-control (IC50 value = 9.80 μM). A similar trend was also observed in C4-2-HOXB3 (IC50 value = 9.12 μM) and C4-2-control (IC50 value = 3.42 μM) (Fig. 2A, D). HOXB3-overexpressed and control CRPC cells were then cultured in medium with or without abiraterone, we found cell proliferation in both control groups were fatally suppressed by adding abiraterone. Nevertheless, imported HOXB3 rescued considerable cell proportions (Fig. 2B, E). For further confirming, we obtained abiraterone resistant C4-2 (C4-2AR) with high HOXB3 protein level (Fig. S1A) and knocked down HOXB3 in them (Fig. S1C). As the results, IC50 value of abiraterone for C4-2AR-shHOXB3 (IC50 value = 5.915 μM) was lower than that of C4-2AR-shScr (IC50 value = 15.539 μM) (Fig. S1D). And, C4-2AR cells re-sensitized to abiraterone after HOXB3 knock down (Fig. S1E). As a CYP17A1 inhibitor, abiraterone plays an important role in inhibiting androgen synthesis and suppressing the expression of the AR-target genes such as PSA and NKX3.1 in CRPC cells. To test if HOXB3 rescue cell survival via A/AR signaling, we examined testosterone and excretive PSA levels in culture medium of transfected LNCaP-AI and C4-2 cells, as well as mRNA expression levels of AR-target genes. Additional HOXB3 had no influence on testosterone generation and AR-target genes expression in tumor cells (Fig. 2C, F-H). Consistent with the proliferation of tumor cells, PSA levels significantly increased in medium cultured HOXB3-overexpressed cells (right panels of the Figs. 2C, F, S1F, G).

To explore the function of HOXB3 in the progression of CRPC in vivo, we analyzed subcutaneous LNCaP-AI xenografts in immunocompromised NOD-SCID mice with or without abiraterone treatment. Tumors arisen from HOXB3-overexpressed LNCaP-AI cells grew significantly faster than those without HOXB3 intervention in mice which received no abiraterone (Fig. 2I, J, crimson vs. gray). Under abiraterone treatment, control LNCaP-AI formed tumors came to standstill until 3 weeks post randomization followed by a gentle recovery, however, xenografts of HOXB3-upregulated LNCaP-AI kept growing at a considerable speed (Fig. 2I, J, blue vs. black), demonstrating that the growth of CRPC tumors depended on HOXB3 when A/AR signaling was blocked. In the whole course of the experiment, HOXB3 remained upregulated in tumor tissues, as confirmed by qRT-PCR of HOXB3 mRNA in harvested tumors (Fig. 2K). The same with LNCaP-AI, we then generated subcutaneous xenografts of C4-2AR-shScr/C4-2AR-shHOXB3 cells in NOD-SCID mice with or without abiraterone treatment. We can see, the most significant tumor inhibition was observed in group with HOXB3 knockdown and abiraterone treatment (Fig. S1H). Together, these results indicated HOXB3 drove CRPC tumor growth and abiraterone resistance independent of A/AR signaling.

### HOXB3 protein level is associated with WNT3A expression in mCRPC

**Fig. 3:**
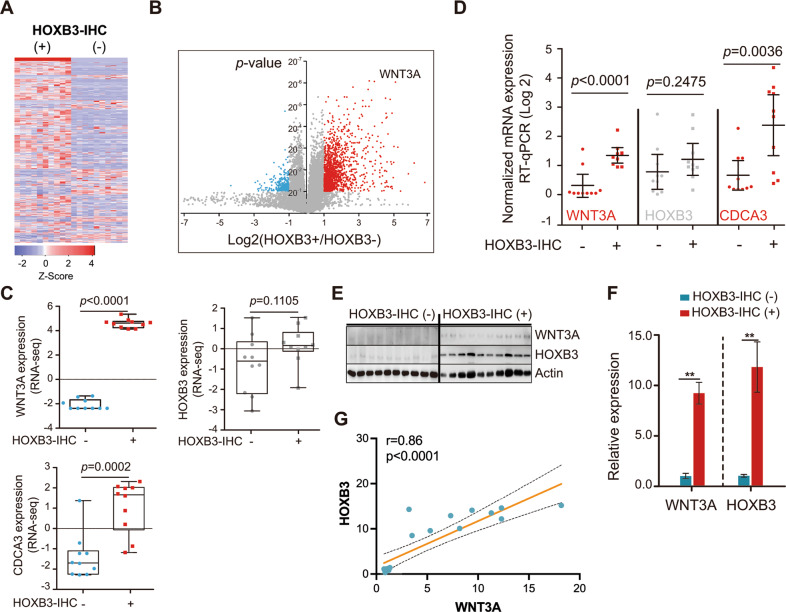
HOXB3 protein, but not mRNA, correlates with WNT3A Expression in PCA. **A** Heatmap showing differentially expressed genes in aPCA with negative (HOXB3−, *n* = 10) and high score (HOXB3+, *n* = 10) of HOXB3 determined by RNA-Seq (see also Table S2). **B** Volume plot showing the significantly different expression of WNT3A between HOXB3+ and HOXB3− tumors determined by RNA-Seq (see also Table S2). **C** Scatter plots with bar showing individual expression of WNT3A, HOXB3, and CDCA3 (target gene of HOXB3) in both HOXB3- and HOXB3 + groups determined by RNA-Seq (see also Table S2). **D** qRT-PCR measured WNT3A, HOXB3, and CDCA3 mRNA expression in both HOXB3− and HOXB3 + groups (*n* = 10 for each group). **E** Western blot of WNT3A, HOXB3, and CDCA3 protein expression in both HOXB3- and HOXB3 + groups (*n* = 10 for each group). Error bars indicate mean ± SD. **F** Quantified results of the WNT3A and HOXB3 protein expression by Image J. **G** The significant correlation between WNT3A and HOXB3 protein expression in Fig. 3E should be determined by Pearson Correlation Coefficient.

Our previous study had shown HOXB3 facilitated PCA progression as a transcription factor partly through transactivating CDCA3 [8]. To better learn how HOXB3 protein was activated and how the activated HOXB3 supported the progression of CRPC, we excised HOXB3- (negative staining in nuclear, *n* = 10) or HOXB3 + (high score staining in nuclear, *n* = 10) tumors and sent them to RNA-seq (Table S2). After standard data processing and quality control, the results indicated that 453 genes were up-regulated, and 36 genes were down-regulated in HOXB3 + tumors (Fig. 3A, Table S3). The activation of WNT pathway is associated with abiraterone resistance [13]. Among the top 20 increased genes, WNT3A was noticed with lowest p value and more than 6.6 folds increment (Fig. 3B). As a transcriptionally regulated gene by HOXB3, the expression of CDCA3 was also higher in HOXB3 + tumors (Fig. 3C), which may indicate HOXB3 was activated in these mCRPC patients. Then, the significantly higher expressions of WNT3A and CDCA3 in HOXB3 + tumors were confirmed by qRT-PCR and western-blotting (Fig. 3D, E), the quantified results of the WNT3A and HOXB3 protein level were shown in Fig. 3F. Significant correlation between WNT3A and HOXB3 protein was determined by Pearson Correlation Coefficient (Fig. 3G). However, only a trend of higher expression of HOXB3 mRNA was detected without significance in RNA-seq and qRT-PCR assays (Fig. 3C, D). The inconsistence between mRNA and protein of HOXB3 indicated that post-transcriptional regulation was essential for its upregulated protein expression. What’s more, HOXB3 knockdown in LNCaP-AI and hCAF (human cancer-associated fibroblasts) cells didn’t change WNT3A expression (data not shown). hCAF cells are primary cultured from the PCA specimens in our laboratory [22]. These results revealed HOXB3 protein level was associated with WNT3A expression, and may be a downstream of WNT3A.

### HOXB3 can be activated by extra WNT3A and the dysfunction of destruction complex

**Fig. 4:**
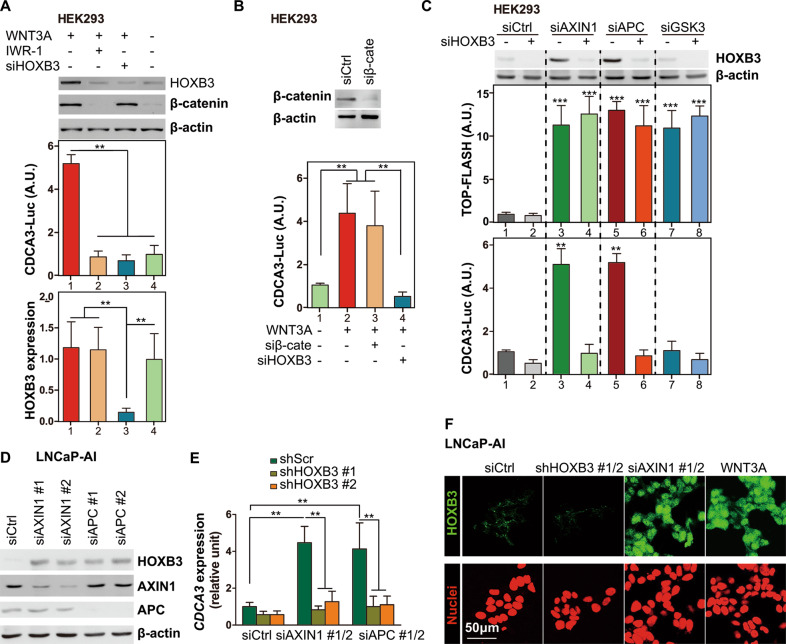
HOXB3 can be activated by extra WNT3A and the dysfunction of destruction complex, see also Fig. S2. **A** HEK293 cells were transfected with CDCA3-Luc and siRNAs for control (siCtrl) or HOXB3 (siHOXB3). Transfected cells were cultured in medium with or without WNT3A and IWR-1 (enhancing AXIN1 stabilization). The results of western blots (for HOXB3, β-catenin and β-actin), luciferase assay (for CDCA3-Luc recording HOXB3-dependent transcriptional activity), and qRT-PCR assay (for HOXB3) were presented. Data were normalized to control (lane 4). **B** HEK293 cells were transfected with CDCA3-Luc and siRNAs (siCtrl, siHOXB3, or siβ-catenin). Transfected cells were cultured in medium with or without WNT3A. The results of western blots (β-catenin and β-actin) and luciferase assay (CDCA3-Luc) of the indicated samples were presented. In luciferase assay, data were normalized to control (lane 1). **C** Top: Western blot of HOXB3 in HEK293 cells as indicated, transfected with siRNAs targeting control (siCtrl), AXIN1 (siAXIN1), APC (siAPC), or GSK3 (siGSK3), followed by another siRNAs targeting control or HOXB3. Bottom: luciferase assays of TOP-FLASH (β-catenin/TCF-dependent transcriptional activity) or CDCA3-Luc reporters in the same cells used for western-blot. **D** Western blots of HOXB3, AXIN1, APC and β-actin in LNCaP-AI cells transfected with siRNAs targeting control (siCtrl), AXIN1 (siAXIN1) or APC (siAPC). **E** qRT-PCR assay of CDCA3 in LNCaP-AI cells after stable transfected by shScr or shHOXB3. **F** Immunofluorescent staining of HOXB3 (green) in LNCaP-AI cells transfected with indicated siRNAs. Nuclei are stained with DAPI (red). Error bars indicate mean ± SD. **p < 0.01, ***p < 0.001.

To verify whether HOXB3 could be activated by WNT3A, exogenous WNT3A was introduced to HEK293 cells. Extra WNT3A triggered significantly increased in both β-catenin and HOXB3 protein (Fig. 4A, top). In addition, WNT3A boosted the initiation of CDCA3-Luc (Fig. 4A, middle), a luciferase reporter containing promoter of CDCA3, the transcription of which had been demonstrated to be regulated by HOXB3 [8]. To the contrary, WNT3A had little effect on HOXB3 mRNA (Fig. 4A, bottom). Besides, the promotion of HOXB3 caused by WNT3A could be abolished by siHOXB3 (a siRNA targeting endogenetic HOXB3) or IWR-1 (a small molecule strengthening the destruction complex via enhancing AXIN1 stabilization) (Fig. 4A). These results suggested that HOXB3 might be stabilized and activated by WNT3A through dispelling destruction complex, like β-catenin and Yes-associated protein 1 (YAP1) [10, 23]. Our conclusion was consistent with hypothesis that the dysfunction of the destruction complex by knockdown AXIN1 or APC induced HOXB3 stabilization and activation (Fig. 4C). Unlike YAP1 protein, deletion of β-catenin or GSK3 had no impact on HOXB3 activation, determined by CDCA3-Luc (Fig. 4B, C), indicating that β-catenin or GSK3 wasn’t necessary for HOXB3-specific destruction complex and WNT3A-induced HOXB3 activity wasn’t transcriptionally regulated by β-catenin/TCF.

To confirm this observation in CRPC cells, LNCaP-AI was chosen to be a cellular model. The reduction of AXIN1 or APC resulted in up-regulation of HOXB3 protein level and CDCA3 mRNA expression, which could be inhibited by knocking down HOXB3 (Fig. 4D, E, and S2A-C). Furthermore, nuclear accumulation of HOXB3 was also increased in LNCaP-AI cells after siAXIN1 or WNT3A treatment (Fig. 4F). Similarly, nuclear accumulation of HOXB3 was decreased in C4-2AR cells when WNT3A was inhibited by IWR-1 (Fig. S2D). Meanwhile, the mRNA expression of CDCA3 was also decreased when C4-2AR was treated with IWR-1 (Fig. S2F). Taken together, these results demonstrated that HOXB3 accumulation and activation could be induced by WNT3A and the dysfunction of destruction complex.

### HOXB3 directly binds to destruction complex

**Fig. 5:**
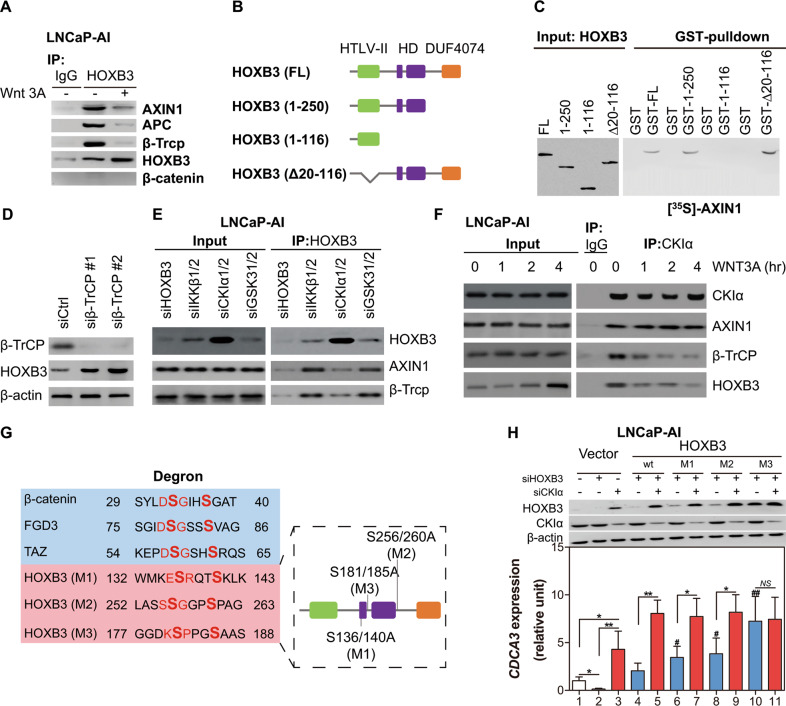
HOXB3 can be degraded by β-TrCP in destruction complex depend on the kinase activity of CKIα, see also Fig. S3. **A** Western blot of indicated proteins CO-IP with HOXB3 in LNCaP-AI cells cultured in medium with or without WNT3A. **B** Graph showing HOXB3 structure with distinguishable functional domains distinguished [24]: HTLV-II domain (N-terminal), Homeobox domain, and DUF4074 domain (C-terminal). **C** Autoradiography of 35S-AXIN1 pulled by GST-fused full-length or truncated HOXB3 as indicated. Input: western blot of GST fused truncated HOXB3 the same as samples used in pull-down experiments. **D** Western blot of β-TrCP and HOXB3 in LNCaP-AI cells treated with siRNAs targeting control (siCtrl) or β-TrCP (siβ-TrCP). **E** Western blot of AXIN1 and β-TrCP CO-IP with HOXB3 in LNCaP-AI cells after deletion of HOXB3, IKKβ, CKIα, or GSK3. Inputs: Western blot of HOXB3, AXIN1 and β-TrCP in the same lysates. **F** Western blot of AXIN1, β-TrCP and HOXB3 CO-IP with CKIα in WNT3A-treated LNCaP-AI cells that harvested at different time points as indicated. Inputs: Western blot of CKIα, HOXB3, AXIN1 and β-TrCP in the same lysates as above. **G** Graph showing phospho-degrons in HOXB3 and other proteins that may be recognized by CKIα, IKKβ, or GSK3. Diagrammatic sketch in frame showing three induced mutants of HOXB3 (M1, M2, and M3 for short) and mutation sites. **H** Top: western blot of HOXB3, CKIα and β-actin in LNCaP-AI cells transfected with siCtrl, siHOXB3 or siCKIα, followed by reconstituting with vector or siRNA-insensitive HOXB3, either wild-type (wt) or its mutants. Cells with S181/185 A mutation became insensitive to CKIα. Bottom: qRT-PCR analyses of CDCA3 expressions in the same cells as above. Error bars indicate mean ± SD. ^*^p < 0.05, ^**^p < 0.01, ^#^*p* < 0.05 relative to line 4, ^##^*p* < 0.01 relative to line 4.

To test if HOXB3 was directly degraded by destruction complex, co-immunoprecipitation (CO-IP) was carried out by targeting HOXB3 in LNCaP-AI cells, which was proved to be a sort of WNT OFF cell line with functional destruction complex. The data showed HOXB3 could bind to the main components of destruction complex: AXIN1, APC and β-TrCP (Fig. 5A). Then, another CO-IP assay was performed targeting AXIN1, the rate-limiting scaffold of the destruction complex, further confirming that AXIN1 could bind to HOXB3 as well as APC, β-TrCP and β-catenin (Fig. S3A). As a core component of WNT pathway, the destruction complex destroyed proteins via direct connection [10, 23]. The direct binding of HOXB3 to AXIN1 was then demonstrated by GST Pull-Down using cell-free recombinant proteins (Fig. S3B).

HOXB3 protein has 3 potential functional domains (Fig. 5B): Human T-cell Leukemia Virus Type II Matrix (HTLV-II, N-terminal), Homeobox domain (HD) and Myeloperoxidase subunit C (DUF4074, C-terminal) [24]. To search the specific domain of HOXB3 binding to AXIN1, GST Pull-Down assay was performed again. The results showed that recombinant fragmentary HOXB3 without HD domain failed in binding to AXIN1 (Fig. 5C). To corroborate this finding in cells, truncated HOXB3 fused with Flag and Myc-AXIN1 were transfected into HEK293 followed by CO-IP experiment. By this way, we found HOXB3 with HD domain was sufficient to bind to AXIN1(Fig. S3C). The above researches supported the view that HOXB3 could directly bind to the destruction complex as one of its components.

### HOXB3 degradation induced by destruction complex requires kinase CKIα

After binding to the destruction complex, targeted proteins of the complex including β-catenin and YAP1, would be degraded by β-TrCP, one of E3 ubiquitin ligases [10, 18]. Similarly, β-TrCP deletion also led to the accumulation of HOXB3 (Fig. 5D). In CO-IP experiments above, we found that extra WNT3A separate β-TrCP from AXIN1 and HOXB3 of the destruction complex (Figs. 5A and S3A). These results supported HOXB3 was a substrate of β-TrCP.

Before being targeted and degraded by β-TrCP, the potential substrates (including β-catenin and YAP1) of β-TrCP needed to be phosphorylated by one or more kinases, such as GSK3, IKKβ and CKIα [23, 25–28]. To explore the required kinases, we respectively knocked down CKIα, GSK3 and IKKβ, and the final data showed a remarkably increased HOXB3 after CKIα deletion and a slightly upregulation after removing IKKβ (Figs. 5E and S3D). In contrast, it was little influenced by GSK3 (Fig. S3D). The following CO-IP assay demonstrated that AXIN1/β-TrCP/HOXB3 complex was significantly weakened after CKIα deletion (Fig. 5E). Moreover, we found that HOXB3 accompanied by β-TrCP gradually kept away from CKIα after WNT3A treatment in LNCaP-AI cells (Fig. 5F). Then, isolated HOXB3 moved into nuclear (Fig. 4F). These results revealed that CKIα played a critical role in the degradation of HOXB3 by destruction complex.

As reported in the literature, kinases phosphorylate β-TrCP substrates within their canonical (DSGxxS sequence) or non-canonical (xSxxxS sequence) phospho-degrons [29, 30]. After seeking for, no canonical degron was detected within HOXB3 (Fig. 5G). To explore if the non-canonical degrons were necessary for HOXB3 degradation, we generated 3 HOXB3 mutants with edited degron (M1, M2 and M3, Fig. 5G). Next, M1, M2, M3 and wild type (wt) HOXB3 were transfected into LNCaP-AI cells after deleting intrinsic HOXB3. The western-blotting and qRT-PCR arrays demonstrated that M3 induces significant increase in HOXB3 and the subsequent CDCA3, while M1 and M2 had limited effect on HOXB3 degradation (Fig. 5H). The results were then confirmed in another CRPC cell line C4-2 (Fig. S3E). At last, we found that M3 successfully prevent HOXB3 from binding to the destruction complex (Fig. S3F). Taken together, our results indicated that CKIα was essential for phosphorylating HOXB3 at non-canonical degron to trigger its destruction.

### HOXB3 transcriptionally drives multiple WNT-regulated genes in PCA

**Fig. 6:**
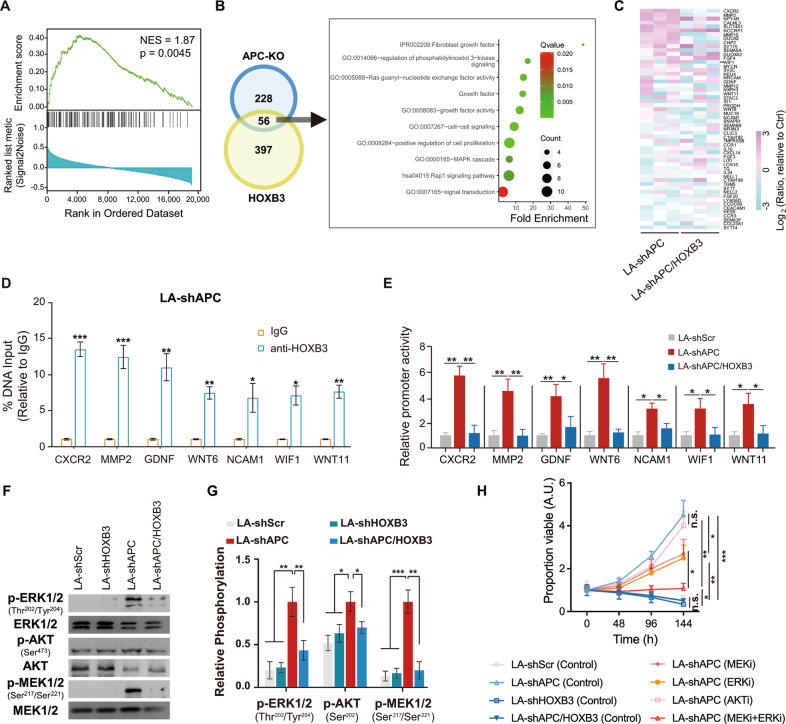
HOXB3 transcriptionally drives multiple WNT-regulated genes and is required for abiraterone resistance in APC-deficient CRPC cells, see also Fig. S4. **A** GSEA of the APC-KO associated genes in data set of GSE144325 comparing differentially expressed genes in HOXB3 high HOXB3 negative tumors. Normalized enrichment score (NES) = 1.87, *p* = 0.0045. **B** Venn diagram showing genes significantly up-regulated in HOXB3 high and APC-KO samples. Overlapped genes showing significant enrichments in cell proliferation determined by Functional Annotation Tool [32], including RAS, PI3K-AKT and MAPK signals (See also Tables S3, S4). **C** LNCaP-AI cells were stably infected by shScramble (short for LA-shScr), shAPC (short for LA-shAPC), or by shAPC and shHOXB3 (short for LA-APC/HOXB3), followed by qRT-PCR assay for the overlapped genes in these cells. Heat maps showing the results of PCR that shAPC upregulated almost all the overlapped genes, while most of them overturned by additional HOXB3 knockdown. **D** qRT-PCRs assay based on CHIP experiments showing HOXB3 binds to the promoters of representative WNT-regulated genes. ChIP experiments using anti-HOXB3 antibody or normal IgG were performed in LA-shAPC cells. **E** The pGL3-CDCA3 was co-transfected with pcDNA3.1-HOXB3 or pcDNA3.1-vector into LA-shScr, LA-shAPC, and LA-APC/HOXB3 cells. Luciferase assays for promoter activities of representative WNT-regulated genes in indicated cells 24 h post transfection. **F**, **G** Western-blot and Bar graph showing upregulated phosphorylation on ERK1/2 (Ras signaling pathway), AKT (PI3K signaling pathway), and MEK1/2 (MAPK signaling pathway) by APC-knockdown in LNCaP-AI cells, which could be overturned by additional HOXB3-knockdown. **H** MTT assays in LNCaP-AI cells infected by shHOXB3, sh-APC or shHOXB3+shAPC (shAPC/HOXB3). All of these cells were cultured in 10% CSS-FBS medium with abiraterone. And with or without specific inhibitors of MEK1/2, ERK1/2, AKT1/2/3. Error bars indicate mean ± SD. *p < 0.05, **p < 0.01, ***p < 0.001.

There is another corollary to the destruction complex regulating HOXB3: some downstream genes of WNT signaling pathway may be transcripts of HOXB3 in WNT-ON cells, such as APC-knockout (APC-KO) PCA cells, a kind of well-studied WNT ON cells with WNT signaling excessive activation [31]. This hypothesis was supported by the GSEA result that revealed HOXB3 associated genes was enriched in dataset [31] of APC-KO vs. APC-intact cells (Fig. 6A). To find the potential HOXB3-transcribed genes, we screened the crossing genes of HOXB3 and WNT by comparing the different expressed genes in HOXB3 + *vs*. HOXB3- tumors and APC-KO *vs*. APC-intact samples (Fig. 6B). Compared with HOXB3- tumors, 453 genes were significantly higher expressed in HOXB3 + ones (Table S3). Meanwhile, a total of 284 genes were notably upregulated in APC-KO samples [31]. As a result, a gene-set containing 56 genes were obtained from the overlap of the two datasets (Table S4). For these 56 genes, cell proliferation (CP) and tumor microenvironment programming (TMP) were identified as the most enriched pathways using Functional Annotation Tool [32] (Figs. 6B, S4A). These results showed that HOXB3 might be a driver of WNT signaling that controlling TMP and CP in CRPC.

To confirm the role of HOXB3 in driving the 56 genes downstream WNT signaling pathway, we introduced shRNAs targeting control (shScr) or APC (shAPC) to LNCaP-AI or C4-2 cells with (shAPC/HOXB3) or without HOXB3 knockdown (Fig. S4B–E). In the subsequent PCR assay, most of the 56 genes (including all genes involved in TMP and CP) were upregulated after APC deletion in LA-shAPC (LNCaP-AI with APC knockdown) cells, but not in LN-shAPC/HOXB3 cells (LNCaP-AI with both APC and HOXB3 knockdown, Fig. 6C). To validate the bind of HOXB3 with the promoter regions of these regulated genes, ChIP assay was performed in LA-shAPC cells. Results showed that activated HOXB3 in LA-shAPC could effectively bind to the promoters of representatives from the 56 genes, including CXCR2, MMP2, GDNF, WNT6, NCAM1, WIF1, and WNT11 (Fig. 6D). To examine the action of HOXB3 was independent of β-catenin-TCF/LEF complex, ChIP-qPCR assay was performed in LA-shAPC cells after knocking down β-catenin or Yap1. The bind of HOXB3 on the promoters of these genes had no significant change when the expression of β-catenin or Yap1 was decreased (Fig. S4F–H). Luciferase report assay showed that the promoter activities of these WNT-genes had significant decreased when HOXB3 was knocked down in C4-2AR cells (Figure S4I). Besides, overexpression of HOXB3 in CRPC cells would not change the protein level of β-catenin (Fig. S4J–K). Further luciferase report assay was also performed in LA-shScr, LA-shAPC and LA-shAPC/HOXB3, suggesting these representative genes are transcriptionally regulated by HOXB3 (Fig. 6E). These results indicated that some WNT-pathway genes are transcriptionally regulated by HOXB3.

### HOXB3 is essential for cell proliferation and abiraterone resistance in APC-deficient CRPC cells

To find out the effects of HOXB3 on the kinase signaling surrounding cell proliferation shown in Fig. 6B, activations of ERK1/2 (RAS signal), AKT (PTEN-PI3K signal) and MEK1/2 (MAPK) were evaluated by Western-blot in LA-Scr (control), LA-shHOXB3 (HOXB3 knockdown), LA-shAPC (APC knockdown) and LA-shAPC/HOXB3 (APC and HOXB3 both knockdown) cells. We found that HOXB3 knockdown was sufficient to impair the robust activation of ERK1/2 and MEK1/2 that induced by APC deficiency in LNCaP-AI cells (Fig. 6F, G). We further asked whether HOXB3 loss was also sufficient to retard APC-deficient induced cell survival and abiraterone resistance. As expected, we found that APC knockdown could effectively increase survival in LNCaP-AI and C4-2 cells even under treatment of abiraterone (Fig. S4L, M). After additional HOXB3 deletion, cell survival significantly reduced, and even completely inhibited in abiraterone treated group (Fig. S4L, M). To confirm the activation of ERK1/2 and MEK1/2 induced by APC deficiency leading to abiraterone resistance, we treated LA-shAPC and C4-2-shAPC cells with the specific inhibitors of MEK1/2, ERK1/2. As results, these specific inhibitors can re-sensitize APC defected CRPC cells to abiraterone (Figs. 6H, S4N). To sum up, the data indicated that HOXB3 was essential for APC-deficient induced cell proliferation and abiraterone resistance in CRPC cells.

### HOXB3 suppression sensitizes WNT-ON CRPC tumor to abiraterone In vivo

**Fig. 7:**
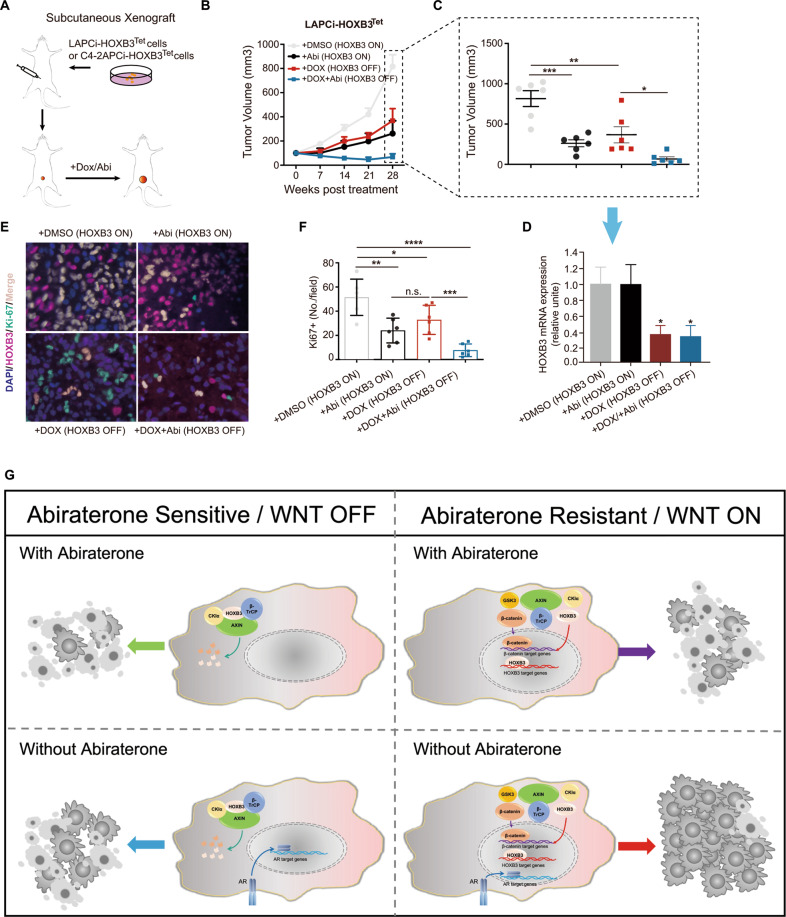
HOXB3 suppression sensitizes APC-defective CRPC xenografts to abiraterone In vivo, see also Fig. S5. **A** Schematic of mouse models of subcutaneous xenografts generated with LNCAP-AI or C4-2 cells stably infected with shAPC and Tet-on shHOXB3. When the tumors size approached approximately 100 mm^3^, mice were randomized to receive DMSO, 0.5 mg/mL Doxycycline (HOXB3 ON), 200 mg/Kg abiraterone, or both (HOXB3 ON). **B**, **C** Caliper measurements were taken weekly for LNCaP-AI xenografts. *n* = 6 mice per group. **B** Line graph with bar showing changes of tumor volumes weekly in each group; (**C**) Scatter plots with bar showing individual tumor volumes at 28 days post-randomization in each group. **D** Bar graph showing HOXB3 stable suppression in HOXB3-OFF LNCaP-AI xenografts at 28 days post-randomization. HOXB3 mRNA levels were measured by qRT-PCR. **E** Immunofluorescent staining of Ki67 (green) and HOXB3 (red) in LNCaP-AI xenografts. Nuclei are stained with DAPI (blue). **F** Scatter plots with bar showing Ki67 expression in each group. **G** Graph abstract depicting the roles of HOXB3 as another downstream effector of the WNT signaling pathway in the WNT ON state. Error bars indicate mean ± SD. *p < 0.05, **p < 0.01, ***p < 0.001.

To research the function of HOXB3 activation in APC-suppressed tumors (one type of WNT-ON tumor), we introduced a doxycycline-inducible shRNA targeting HOXB3 (Tet-off) into the LNCaP-AI cells with APC knockdown (LAPCi-HOXB3^Tet^ for short). When tumors approached a size of approximately 100mm^3^ in NOD-SCID mice, xenografts were randomized to receive water without doxycycline plus vehicle (HOXB3 ON), water with doxycycline plus vehicle (HOXB3 OFF), water without doxycycline plus abiraterone (HOXB3 ON) or water with doxycycline plus abiraterone (HOXB3 OFF), respectively (Fig. 7A). The fastest growth rate of tumor was detected in xenografted mice that received neither doxycycline nor abiraterone (Fig. 7B, C, gray). The growth rates of HOXB3-ON xenografts were reduced by abiraterone or doxycycline-induced HOXB3-konckdown, however, tumor progressions never stopped in the two groups (Fig. 7B, C, black and crimson). Notably, abiraterone could cause a curative treatment in HOXB3-OFF tumors (Fig. 7B, C, blue). In addition, levels of HOXB3 mRNA were evaluated by qRT-PCR at the end of this experiment, showing that HOXB3 remained suppressed in HOXB3-OFF tumor tissues (Fig. 7D). At last, the results were confirmed in C4-2 cells with APC-knockdown (Fig. S5A–C). Xenograft tumor still grew with a slow speed under abiraterone treatment, indicating WNT-ON CRPC tumor is relatively resistant to abiraterone. However, abiraterone and HOXB3 suppression could significantly decrease the proliferation of WNT-ON CRPC tumor (Fig. 7E, F and S5D). Altogether, these results indicated HOXB3 drove WNT-associated tumor progression and abiraterone resistance in CRPC xenograft, and antiandrogen combined with HOXB3 suppression could be potential therapy for CRPC with WNT-pathway activation (Fig. 7G).

## Discussion

The mechanisms that enable CRPC cells to progress and weaken NHT are understood insufficiently. As mentioned above, our study shows that HOXB3 is an independent risk factor of PSA progression and death in mCRPC patients. To be specific, HOXB3 serves as a downstream transcription factor of WNT pathway and defines a subtype of CRPC with WNT activation that resistant to antiandrogen. Finally, HOXB3 suppression reduces cell proliferation in WNT-ON CRPC cells and sensitizes WNT-ON CRPC tumors to abiraterone. These results revealed a link between HOXB3 and WNT signaling, molded a type of NHT-resistant CRPC, and identified HOXB3 as a therapeutic target of WNT-ON CRPC.

### WNT and prostate cancer progression and therapy-resistance

WNT signal is a general designation of canonical WNT signaling, noncanonical WNT signaling, and WNT-dependent stabilization of proteins (WNT-STOP), a novel transcription-independent branch of WNT signal that is initiated by WNT binding to LRP6 [33]. Previous studies have shown that WNT-pathway activation is associated with higher Gleason grades and PSA levels [13]. In addition, aberrant activation of the WNT pathway is causative to multiple cancer progression, which may be caused by mutations of pathway components or dyshomeostasis of autocrine/paracrine signaling [31, 34, 35]. Among them, functional mutations in WNT pathway components have been demonstrated to be important for progression and NHT resistance in mCRPC patients.

There are about 10–20% advanced PCA patients harbored alterations in the WNT signaling pathway, including inactivating mutations in APC and RNF43, and activating mutations in CTNNB1 and RSPO2 [13, 16]. It’s worth noting that activated WNT reach up to 71% in advanced PCA using immunohistochemistry [36]. In other word, upstream components of the pathway may be responsible for the high frequency of WNT signal activation besides somatic mutations. Emerging evidence show WNT3A [37, 38], WNT16B [39] and WNT5A [34] are essential in PCA for its initial, development, progression and therapy resistance, which may be autocrine or paracrine from stroma. In this study, higher WNT3A was detected in HOXB3 + tumors and was associated with higher tumor grade and stage. In according with reported percentage of WNT activation in advanced PCA [36], medium to high nuclear staining of HOXB3 was 57% in this work.

### Differential regulation of HOXB3, β-catenin and YAP1 by WNT

Target genes of β-catenin, such as SOX9 and MYC, can also mediate WNT signaling [40]. Here’s the difference that, HOXB3 can’t be transcriptionally regulated by β-catenin (Fig. 4A). This work discovered an alternative WNT signaling pathway mediated by HOXB3. WNT3A mobilizes β-catenin, YAP1 and HOXB3 utilizing similar way but not sharing the same mechanism. Before being degraded by destruction complex, phosphorylation by GSK3 on phospho-degron is necessary for β-catenin and YAP1, while YAP1 and HOXB3 rely on CKIα [23]. Without phosphorylation in phospho-degrons, these effectors would be accumulated in cells and translocated into nuclei.

There are some crosstalks among the three sub-pathways that may maintain the WNT signaling homeostasis in cells. YAP1 can be activated by both WNT3A and WNT5A/B. It’s interesting to note that WNT5A/B are also downstream target genes of YAP1, which indicates a potential positive feedback loop [41]. Target genes of YAP1 also contain DKK1, which plays a role in inhibiting WNT/β-catenin/TCF. However, the accumulation and activation of YAP1 are insensitive to WNT5A/B, DKK1 and β-catenin deletion [41, 42]. In turn, studies showed YAP1 might be negatively regulated by HOXB3. In Intervertebral disc, overexpressed DNA Methyltransferase 3 Beta (DNMT3B) could effectively inhibit COX2/YAP1 axis via modifying the promotor of transient receptor potential ankyrin 1 (TRPA1) by methylation [43]. In addition to this, HOXB3 was a direct activator of DNMT3B promoting its transcription [6]. In conclusion, these results could support our hypothesis that WNT homeostasis was sustained by HOXB3, β-catenin and YAP1 via interactively positive and negative feedback loops.

### HOXB3, an essential effector of WNT Signaling

β-catenin is a well-known canonical effector of WNT signaling pathway. Moreover, YAP1 has been confirmed to be an alternative mediator of WNT pathway that can be also degraded by the destruction complex [23, 41]. One hypothesis was proposed that other factors, such as HOXB3, might also contribute to WNT induced biological functions. In this work, HOXB3 was proved to be another mediator of WNT signaling based on its close relation to WNT3A observed in clinical mCRPC samples. The activations of these effectors would depend on the requirements of specific cells, which was known inadequately. Sometimes, the biological effects of WNT might primarily depend on one of the effectors, however, previous studies more likely supported that there would be more than one effector cooccurring in response to WNT ligands [18, 23]. For example, in colorectal cancer (CRC), β-catenin and YAP1 complementarily play critical roles on renewal and differentiation of mesenchymal stem cells, proliferation potential are the cooperative function of YAP1 and HOXB3 that isn’t shared by β-catenin, while all of them contribute to cells’ migration and invasion [6, 23, 41, 44].

In PCA, YAP1 only exist in basal/dedifferentiated PCA and stroma cells with little androgen receptor (AR) signaling activation, which contribute to the development of anti-androgen resistance caused by induction of cancer stemness [45, 46]. Interestingly, YAP1 would disappear again after the PCA cells move forward to acquiring an aggressive neuroendocrine (NE) phenotype [47, 48]. What’s more, in this study, we found that some classical NE-markers (e.g., CHGA and NCAM1) significantly increased in HOXB3 positive cells, indicating HOXB3 might be a promotor of NE differentiation that needed further confirmation. HOXB3 and β-catenin are both responsible for migration and invasion [2]. While PCA proliferation is more likely sustained by HOXB3 [8].

## Conclusions

Our study establishes the connection between HOXB3 and WNT pathway activation in CRPC from a fresh perspective and mechanistically shows that HOXB3 positively regulates multiple genes of WNT signaling. WNT-HOXB3 activation may be dependent on autocrine or paracrine WNT ligands or be enhanced by mutations in key components of the WNT signaling pathway. Upregulated HOXB3 results in progression and antiandrogens resistance in at least a subset CRPC tumors with WNT pathway activation, and this subgroup of antiandrogen-insensitive CRPC will benefit from antiandrogens therapy combined with HOXB3 targeting.

## Supplementary information

Supplementary materials (Tables S1 X S5, Fgures S1-S5)

Table S2

Table S3

Table S4

Checklist

Authorship Confirmation

Original Data File

## Data Availability

The RNA-seq data have been deposited in supplementary materials. The datasets generated and/or analysed during the current study are available from the corresponding author on reasonable request.

## Code availability

All R codes used in this study are available by contacting SZ.

## References

[1] de Bono JS, Logothetis CJ, Molina A, Fizazi K, North S, Chu L (2011). Abiraterone and increased survival in metastatic prostate cancer. N Engl J Med.

[2] Murillo-Garzon V, Kypta R (2017). WNT signalling in prostate cancer. Nat Rev Urol.

[3] Schworer S, Becker F, Feller C, Baig AH, Kober U, Henze H (2016). Epigenetic stress responses induce muscle stem-cell ageing by Hoxa9 developmental signals. Nature.

[4] Malek R, Gajula RP, Williams RD, Nghiem B, Simons BW, Nugent K (2017). TWIST1-WDR5-Hottip regulates Hoxa9 chromatin to facilitate prostate cancer metastasis. Cancer Res.

[5] Sio A, Chehal MK, Tsai K, Fan X, Roberts ME, Nelson BH (2013). Dysregulated hematopoiesis caused by mammary cancer is associated with epigenetic changes and hox gene expression in hematopoietic cells. Cancer Res.

[6] Palakurthy RK, Wajapeyee N, Santra MK, Gazin C, Lin L, Gobeil S (2009). Epigenetic silencing of the RASSF1A tumor suppressor gene through HOXB3-mediated induction of DNMT3B expression. Mol Cell.

[7] Yu Z, Liu J, Fan Q, Yu J, Ren X, Wang X (2022). Extracellular vesicle-encapsulated microRNA-375 from bone marrow-derived mesenchymal stem cells inhibits hepatocellular carcinoma progression through regulating HOXB3-mediated Wnt/beta-catenin pathway. Anal Cell Pathol (Amst).

[8] Chen J, Zhu S, Jiang N, Shang Z, Quan C, Niu Y (2013). HoxB3 promotes prostate cancer cell progression by transactivating CDCA3. Cancer Lett.

[9] Nusse R, Clevers H (2017). Wnt/beta-catenin signaling, disease, and emerging therapeutic modalities. Cell.

[10] MacDonald BT, Tamai K, He X (2009). Wnt/beta-catenin signaling: components, mechanisms, and diseases. Dev Cell.

[11] Barolo S (2006). Transgenic Wnt/TCF pathway reporters: all you need is Lef?. Oncogene.

[12] Niehrs C, Acebron SP (2012). Mitotic and mitogenic Wnt signalling. EMBO J.

[13] Isaacsson Velho P, Fu W, Wang H, Mirkheshti N, Qazi F, Lima FAS (2020). Wnt-pathway activating mutations are associated with resistance to first-line abiraterone and enzalutamide in castration-resistant prostate cancer. Eur Urol.

[14] Liu Z, Wang L, Zhou Y, Wang C, Ma Y, Zhao Y (2021). Application of metastatic biopsy based on “when, who, why, where, how (4W1H)” principle in diagnosis and treatment of metastatic castration-resistance prostate cancer. Transl Androl Urol.

[15] Zhu S, Tian H, Niu X, Wang J, Li X, Jiang N (2019). Neurotensin and its receptors mediate neuroendocrine transdifferentiation in prostate cancer. Oncogene.

[16] Robinson D, Van Allen EM, Wu YM, Schultz N, Lonigro RJ, Mosquera JM (2015). Integrative clinical genomics of advanced prostate cancer. Cell.

[17] Trapnell C, Roberts A, Goff L, Pertea G, Kim D, Kelley DR (2012). Differential gene and transcript expression analysis of RNA-seq experiments with TopHat and Cufflinks. Nat Protoc.

[18] Azzolin L, Panciera T, Soligo S, Enzo E, Bicciato S, Dupont S (2014). YAP/TAZ incorporation in the beta-catenin destruction complex orchestrates the Wnt response. Cell.

[19] Shang Z, Yu J, Sun L, Tian J, Zhu S, Zhang B (2019). LncRNA PCAT1 activates AKT and NF-kappaB signaling in castration-resistant prostate cancer by regulating the PHLPP/FKBP51/IKKalpha complex. Nucleic Acids Res.

[20] Chai J, Du C, Wu JW, Kyin S, Wang X, Shi Y (2000). Structural and biochemical basis of apoptotic activation by Smac/DIABLO. Nature.

[21] Redelsperger IM, Taldone T, Riedel ER, Lepherd ML, Lipman NS, Wolf FR (2016). Stability of doxycycline in feed and water and minimal effective doses in tetracycline-inducible systems. J Am Assoc Lab Anim Sci.

[22] Shen T, Li Y, Zhu S, Yu J, Zhang B, Chen X (2020). YAP1 plays a key role of the conversion of normal fibroblasts into cancer-associated fibroblasts that contribute to prostate cancer progression. J Exp Clin Cancer Res: CR.

[23] Azzolin L, Zanconato F, Bresolin S, Forcato M, Basso G, Bicciato S (2012). Role of TAZ as mediator of Wnt signaling. Cell.

[24] Lewis TE, Sillitoe I, Andreeva A, Blundell TL, Buchan DW, Chothia C (2015). Genome3D: exploiting structure to help users understand their sequences. Nucleic Acids Res.

[25] Zhou BP, Deng J, Xia W, Xu J, Li YM, Gunduz M (2004). Dual regulation of Snail by GSK-3beta-mediated phosphorylation in control of epithelial-mesenchymal transition. Nat Cell Biol.

[26] Winston JT, Strack P, Beer-Romero P, Chu CY, Elledge SJ, Harper JW (1999). The SCFbeta-TRCP-ubiquitin ligase complex associates specifically with phosphorylated destruction motifs in IkappaBalpha and beta-catenin and stimulates IkappaBalpha ubiquitination in vitro. Genes Dev.

[27] Taelman VF, Dobrowolski R, Plouhinec JL, Fuentealba LC, Vorwald PP, Gumper I (2010). Wnt signaling requires sequestration of glycogen synthase kinase 3 inside multivesicular endosomes. Cell.

[28] Elyada E, Pribluda A, Goldstein RE, Morgenstern Y, Brachya G, Cojocaru G (2011). CKIalpha ablation highlights a critical role for p53 in invasiveness control. Nature.

[29] Inuzuka H, Tseng A, Gao D, Zhai B, Zhang Q, Shaik S (2010). Phosphorylation by casein kinase I promotes the turnover of the Mdm2 oncoprotein via the SCF(beta-TRCP) ubiquitin ligase. Cancer Cell.

[30] Zhong J, Ogura K, Wang Z, Inuzuka H (2013). Degradation of the transcription factor Twist, an oncoprotein that promotes cancer metastasis. Discov Med.

[31] van Neerven SM, de Groot NE, Nijman LE, Scicluna BP, van Driel MS, Lecca MC (2021). Apc-mutant cells act as supercompetitors in intestinal tumour initiation. Nature.

[32] Sherman BT, Hao M, Qiu J, Jiao X, Baseler MW, Lane HC (2022). DAVID: a web server for functional enrichment analysis and functional annotation of gene lists (2021 update). Nucleic Acids Res.

[33] Acebron SP, Karaulanov E, Berger BS, Huang YL, Niehrs C (2014). Mitotic wnt signaling promotes protein stabilization and regulates cell size. Mol Cell.

[34] Ma F, Arai S, Wang K, Calagua C, Yuan AR, Poluben L (2022). Autocrine canonical Wnt signaling primes noncanonical signaling through ROR1 in metastatic castration-resistant prostate cancer. Cancer Res.

[35] Scheel C, Eaton EN, Li SH, Chaffer CL, Reinhardt F, Kah KJ (2011). Paracrine and autocrine signals induce and maintain mesenchymal and stem cell states in the breast. Cell.

[36] Yardy GW, Bicknell DC, Wilding JL, Bartlett S, Liu Y, Winney B (2009). Mutations in the AXIN1 gene in advanced prostate cancer. Eur Urol.

[37] Nandana S, Tripathi M, Duan P, Chu CY, Mishra R, Liu C (2017). Bone metastasis of prostate cancer can be therapeutically targeted at the TBX2-WNT signaling axis. Cancer Res.

[38] Bhattacharyya S, Feferman L, Tobacman JK (2019). Dihydrotestosterone inhibits arylsulfatase B and Dickkopf Wnt signaling pathway inhibitor (DKK)-3 leading to enhanced Wnt signaling in prostate epithelium in response to stromal Wnt3A. Prostate.

[39] Sun Y, Campisi J, Higano C, Beer TM, Porter P, Coleman I (2012). Treatment-induced damage to the tumor microenvironment promotes prostate cancer therapy resistance through WNT16B. Nat Med.

[40] Ma F, Ye H, He HH, Gerrin SJ, Chen S, Tanenbaum BA (2016). SOX9 drives WNT pathway activation in prostate cancer. J Clin Investig.

[41] Park HW, Kim YC, Yu B, Moroishi T, Mo JS, Plouffe SW (2015). Alternative Wnt signaling activates YAP/TAZ. Cell.

[42] Seo E, Basu-Roy U, Gunaratne PH, Coarfa C, Lim DS, Basilico C (2013). SOX2 regulates YAP1 to maintain stemness and determine cell fate in the osteo-adipo lineage. Cell Rep.

[43] Luo Z, Ma Y, Di T, Ma B, Li H, An J (2021). DNMT3B decreases extracellular matrix degradation and alleviates intervertebral disc degeneration through TRPA1 methylation to inhibit the COX2/YAP axis. Aging (Albany NY).

[44] Almeida MI, Nicoloso MS, Zeng L, Ivan C, Spizzo R, Gafa R (2012). Strand-specific miR-28-5p and miR-28-3p have distinct effects in colorectal cancer cells. Gastroenterology.

[45] Jiang N, Ke B, Hjort-Jensen K, Iglesias-Gato D, Wang Z, Chang P (2017). YAP1 regulates prostate cancer stem cell-like characteristics to promote castration resistant growth. Oncotarget.

[46] Lee HC, Ou CH, Huang YC, Hou PC, Creighton CJ, Lin YS (2021). YAP1 overexpression contributes to the development of enzalutamide resistance by induction of cancer stemness and lipid metabolism in prostate cancer. Oncogene.

[47] Cheng S, Prieto-Dominguez N, Yang S, Connelly ZM, StPierre S, Rushing B (2020). The expression of YAP1 is increased in high-grade prostatic adenocarcinoma but is reduced in neuroendocrine prostate cancer. Prostate Cancer Prostatic Dis.

[48] Pearson JD, Huang K, Pacal M, McCurdy SR, Lu S, Aubry A (2021). Binary pan-cancer classes with distinct vulnerabilities defined by pro- or anti-cancer YAP/TEAD activity. Cancer Cell.

