# Supplementary material for: HOXB3 drives WNT-activation associated progression in castration-resistant prostate cancer
Source: Cell Death Dis. 2023 Mar 27;14(3):215. doi: 10.1038/s41419-023-05742-y (PMC10042887; doi:10.1038/s41419-023-05742-y)
Supplement: Supplementary file 1 — Supplementary materials (Tables S1 X S5, Fgures S1-S5) [file 41419_2023_5742_MOESM1_ESM.pdf]

# Title: HOXB3 drives WNT-activation associated progression in castration-resistant prostate cancer

Shimiao Zhu[1], Zhao Yang[1], Zheng Zhang[1], Hongli Zhang[1], Songyang Li[1], Tao Wu[2], Xuanrong Chen[1], Aixiang Wang[3], Jianing Guo[4], Hao Tian[1], Jianpeng Yu[1], Changwen Zhang[1], Zhiqun Shang[1,**] , Changyi Quan[1,*], Yuanjie Niu[1,***]

## Supplementary Materials

# Contents

**Table S1.** Baseline characteristics of 58 mCRPC patients with HOXB3 assessed by IHC

**Table S2.** RNA-seq data from HOXB3+ and HOXB3- mCRPC

**Table S3.** Differently expressed genes in HOXB3 + vs. HOXB3- mCRPC

**Table S4.** Overlap genes of HOXB3+ vs. HOXB3- and APC-KO vs. APC-intact samples

**Table S5.** Primers used in this study (from primer bank or designed using primer 5)

**Figure S1.** HOXB3 promotes CRPC resistance to abiraterone in vitro and in vivo, related to Figure 2.

**Figure S2.** HOXB3 can be transactivated and stabilized by extra WNT3A and dysfunction of destruction complex, related to Figure 4.

**Figure S3.** HOXB3 can be degraded by β-TrCP in destruction complex depend on the kinase activity of CKIα, related to Figures 5.

**Figure S4.** HOXB3 is an effector of APC-defect driven resistance to novel hormonal therapy in CRPC, related to Figures 6.

**Figure S5.** HOXB3 suppression sensitizes APC-defective CRPC xenografts to abiraterone In vivo, related to Figures 7.

**Table S1.** Baseline characteristics of 58 mCRPC patients with HOXB3 assessed by IHC

| Nuclear Staining | Negative | Low | Medium | | High | | |
|---|---|---|---|---|---|---|---|
| Intensity | None | Weak | Weak | Moderate | Moderate | Strong | |
| Quantity | 0 | <25% | ⩾25% | <25% | ⩾25% | Any | *p* |
| | **n=10** | **n=21** | **n=8** | **n=9** | **n=7** | **n=3** | |
| Age | | | | | | | 0.82‡ |
| <65y | 4 (40%) | 13 (62%) | 7 (41%) | | 7 (70%) | | |
| ≥65y | 6 (60%) | 8 (38%) | 10 (59%) | | 3 (30%) | | |
| iPSA (ng/ml) | | | | | | | |
| Median | 23.2 | 27.3 | 24.7 | | 37.9 | | 0.25† |
| Range | (6.3-103.5) | (2.2-355.8) | (4.2-178.3) | | (1.3-370.1) | | |
| PSA before Abi | | | | | | | |
| Median | 12.5 | 28.9 | 37.2 | | 78.9 | | 0.08† |
| Range | (2.3-190.3) | (2.6-1102.5) | (3.3-2369.4) | | (7.3-1571.0) | | |
| ISUP Grade at initial diagnosis | | | | | | | 0.16‡ |
| ≤3 | 6 (60%) | 10 (48%) | 7 (41%) | | 2 (20%) | | |
| ≥4 | 4 (40%) | 11 (52%) | 10 (59%) | | 8 (80%) | | |
| Visceral disease | | | | | | | 0.04‡ |
| Yes | 0 (0%) | 2 (10%) | 3 (18%) | | 5 (50%) | | |
| No | 10 (100%) | 19 (90%) | 14 (82%) | | 5 (50%) | | |
| Previous therapy | | | | | | | |
| RP/RTx | 3 (30%) | 7 (33%) | 5 (29%) | | 2 (20%) | | 0.60‡ |

| | | | | | |
|---|---|---|---|---|---|
| ADT | 10 (100%) | 21 (100%) | 17 (100%) | 10 (100%) | 1.00‡ |
| Chemotherapy | 1 (10%) | 2 (10%) | 3 (18%) | 3 (30%) | 0.34‡ |
| Baseline HGB (g/dL) | | | | | 0.41† |
| Median | 11.5 | 11.3 | 11.2 | 10.2 | |
| Range | (7.2-16.3) | (7.3-15.8) | (7.1-16.4) | (7.1-14.2) | |
| Baseline LDH | | | | | 0.15† |
| Median | 221 | 226 | 229 | 242 | |
| Range | (89-2356) | (113-3129) | (102-4429) | (221-5031) | |

PSA, initial PSA; Abi, Abiraterone; HGB, hemoglobin; LDH, lactate dehydrogenase

† Student's t test (Negative/Low vs. Medium/High)

‡ Chi-square test (Negative/Low vs. Medium/High)

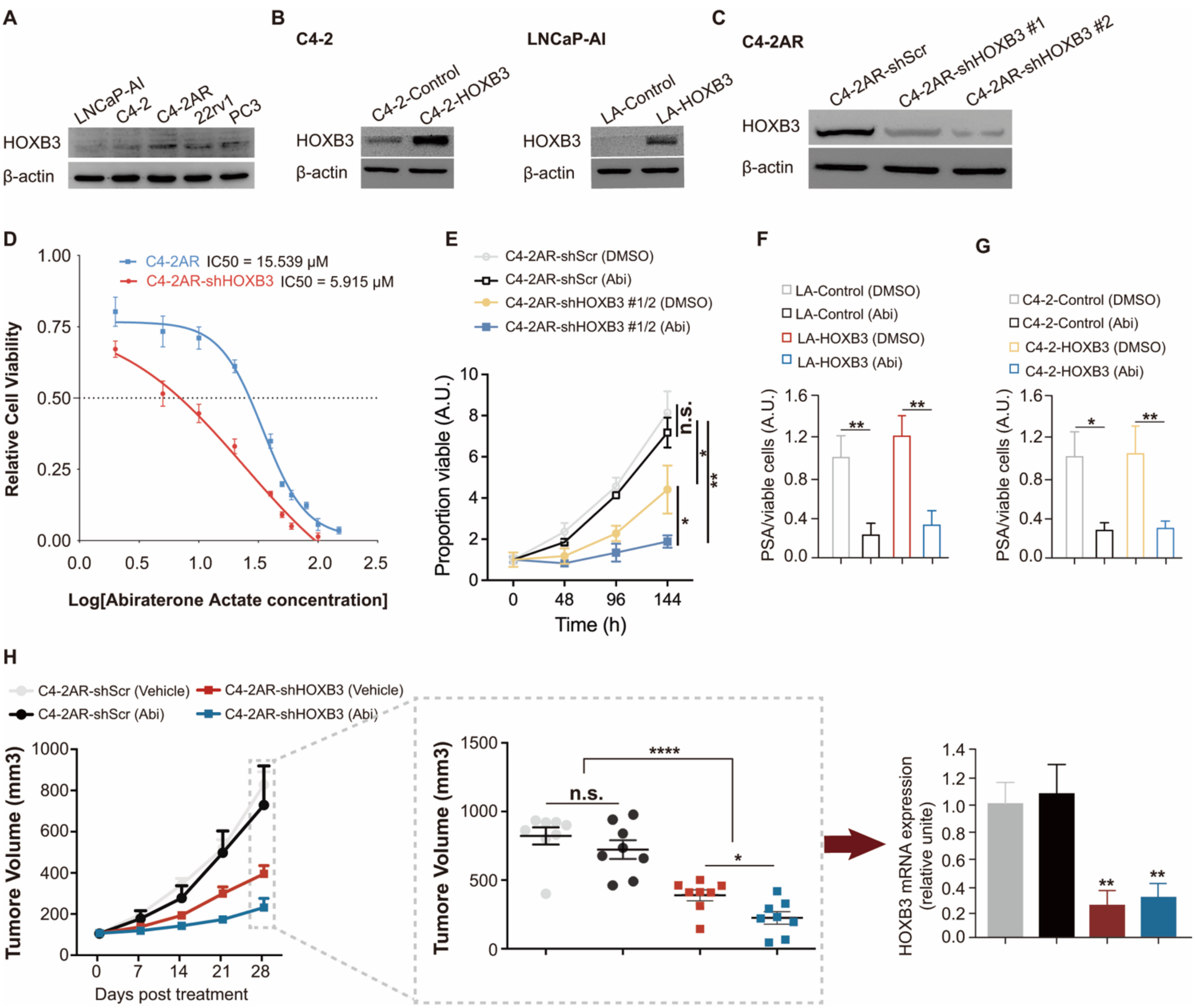


**Figure S1.** HOXB3 promotes CRPC resistance to abiraterone in vitro and in vivo, related to Figure 2.

(**A**) Western blots of HOXB3 in several CRPC cell lines. (**B**) HOXB3 overexpression in LNCaP-AI and C4-2 cells. HOXB3 protein expression was measured by Western blot in LA-HOXB3 vs. LA-Control and C4-2-HOXB3 vs. C4-2-Control. (**C**) HOXB3 konckdown in C4-2AR. Western blots of HOXB3 in C4-2AR-shScr vs. C4-2AR-shHOXB3. (**D**) Cell growth in the presence of different concentrations of abiraterone actate was measured in C4-2AR-shScr/C4-2AR-shHOXB3. IC50 values are indicated by dotted lines. (**E**) MTT assays were performed in C4-2AR-shScr/C4-2AR-shHOXB3 cells after treated with DMSO or 10 μmol/L abiraterone (Abi) for indicated time. (**F and G**) LA-Control/LA-HOXB3 and C4-2-Control/C4-

2-HOXB3 cells were treated with DMSO or 10 μmol/L Abi in charcoal stripped serum for 96 hours. Fifty million cells were collected from each group followed by PSA value test by ELISA. (**H**) Left: C4-2AR-shScr or C4-2AR-shHOXB3 cells were injected subcutaneously in mice and grown until tumors reached a size of ~100 mm3. Xenografted mice were then randomly treated with vehicle control or abiraterone acetate (200 mg/Kg, p.o). Caliper measurements were taken weekly. n = 8 mice per group. Middle: Scatter plots with bar showing individual tumor volumes at 28 days post-randomization in each group (n = 8 per group). Right: Bar graph showing HOXB3 stable suppression in indicated tumors at 28 days post-randomization. HOXB3 mRNA levels were measured by qRT-PCR. Error bars indicate mean ± SD. **represents $p < 0.01$

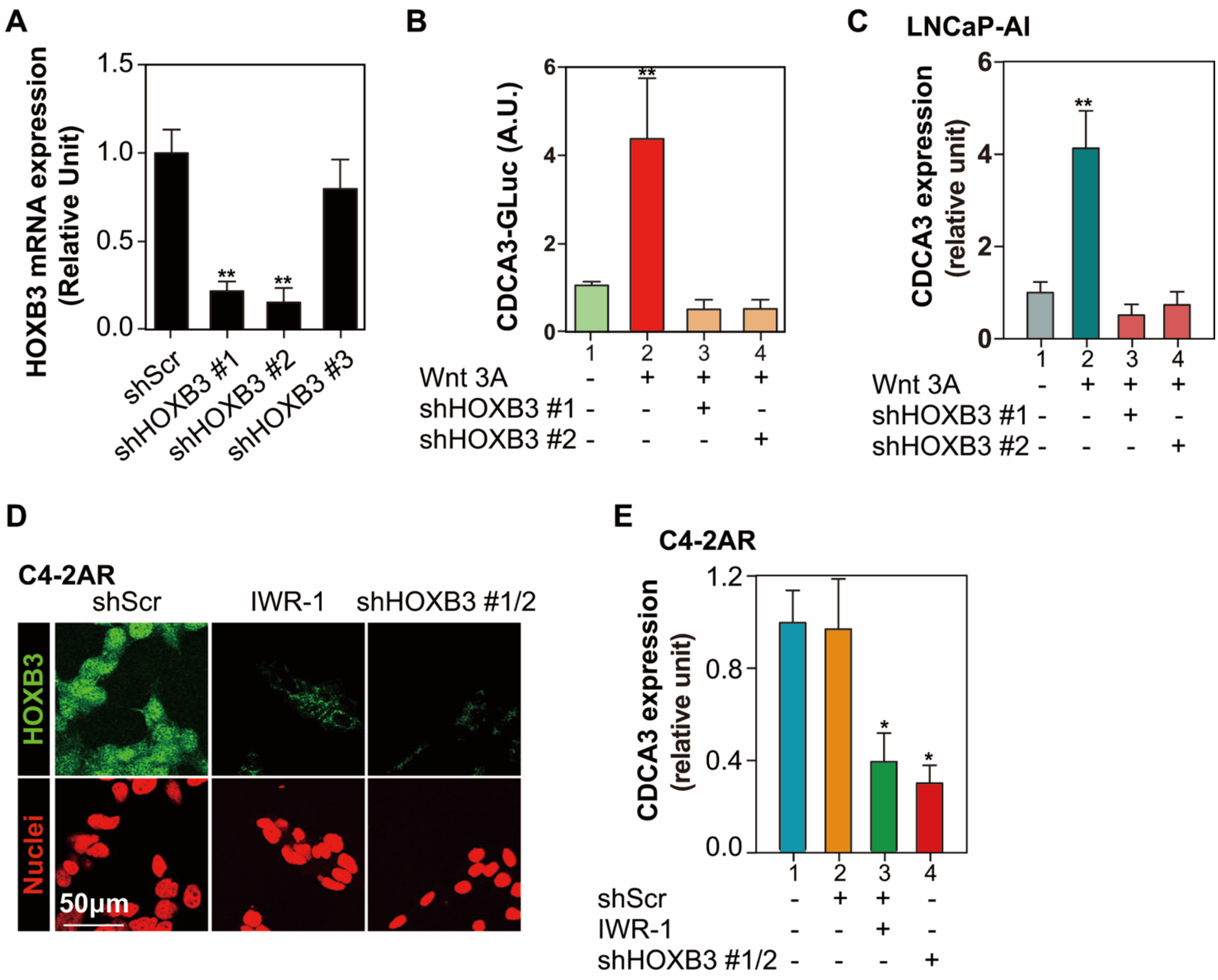


**Figure S2.** HOXB3 can be transactivated and stabilized by extra WNT3A and dysfunction of destruction complex, related to Figure 4.

(**A**) HOXB3 knockdown in LNCaP-AI cells. HOXB3 mRNA was measured by qRT-PCR after infected by shScr (control shRNA) or shHOXB3. (**B**) LNCaP-AI cells were transfected with CDCA3-Luc and shScr/shHOXB3. Stable cells were cultured in medium with or without WNT3A. Luciferase assays in the indicated samples. Data were normalized to control (lane 1). (**C**) RT-qPCR assay of CDCA3 mRNA expression in the same cells indicated above. (**D**) Immunofluorescent staining of HOXB3 (green) in C4-2AR cells treated as indicated. Nuclei are stained with DAPI (red). (**E**) RT-qPCR assay of CDCA3 mRNA expression in C4-2AR cells treated as indicated. Error bars indicate mean ± SD. **represents $p < 0.01$

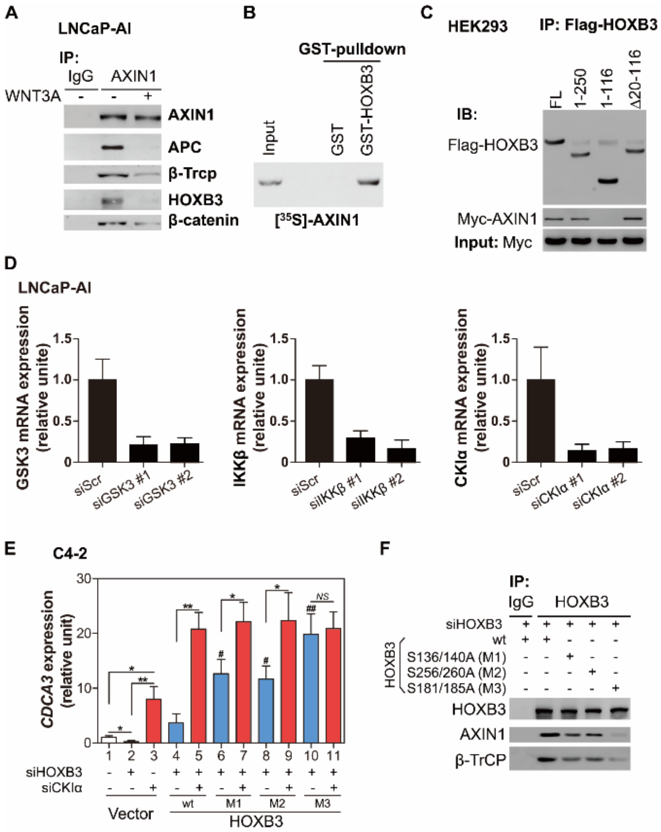


**Figure S3.** HOXB3 can be degraded by β-TrCP in destruction complex depend on the kinase activity of CKIα, related to Figures 5.

(**A**) CO-IP assay of indicated proteins that binding with AXIN1 in LNCaP-AI cells cultured in medium with or without WNT3A. (**B**) Autoradiography of GST-HOXB3 pulled 35S-AXIN1. GST protein and 35S-Axin1 were respectively used as negative control and input. (**C**) CO-IP assay of Myc (fused with AXIN1) that binding with indicated HOXB3 mutants (fused with Flag) in HEK293 cells that transfected with Myc-AXIN1 and indicated Flag-HOXB3. Input: western blot of Myc fused AXIN1 using anti-Myc. (**D**) qRT-PCR analyses of of GSK3, IKKβ and CKIα in LNCaP-AI cells treated with siRNAs targeting control (siCtrl) or indicated kinases. (E) qRT-PCR analyses of CDCA3 expressions in C4-2 cells transfected with siCtrl, siHOXB3 or siCKIα, followed by reconstituting with vector or siRNA-insensitive HOXB3, either wt or mutants. (F) Western blot of AXIN1 and β-TrCP CO-IP with HOXB3 in LNCaP-AI cells treated with siHOXB3, followed by reconstituting with vector or siRNA-insensitive HOXB3, either wt or mutants. Error bars indicate mean ± SD. *represents $p < 0.05$, **represents $p < 0.01$, #represents $p < 0.05$ relative to line 4, ##represents $p < 0.01$ relative to line 4.

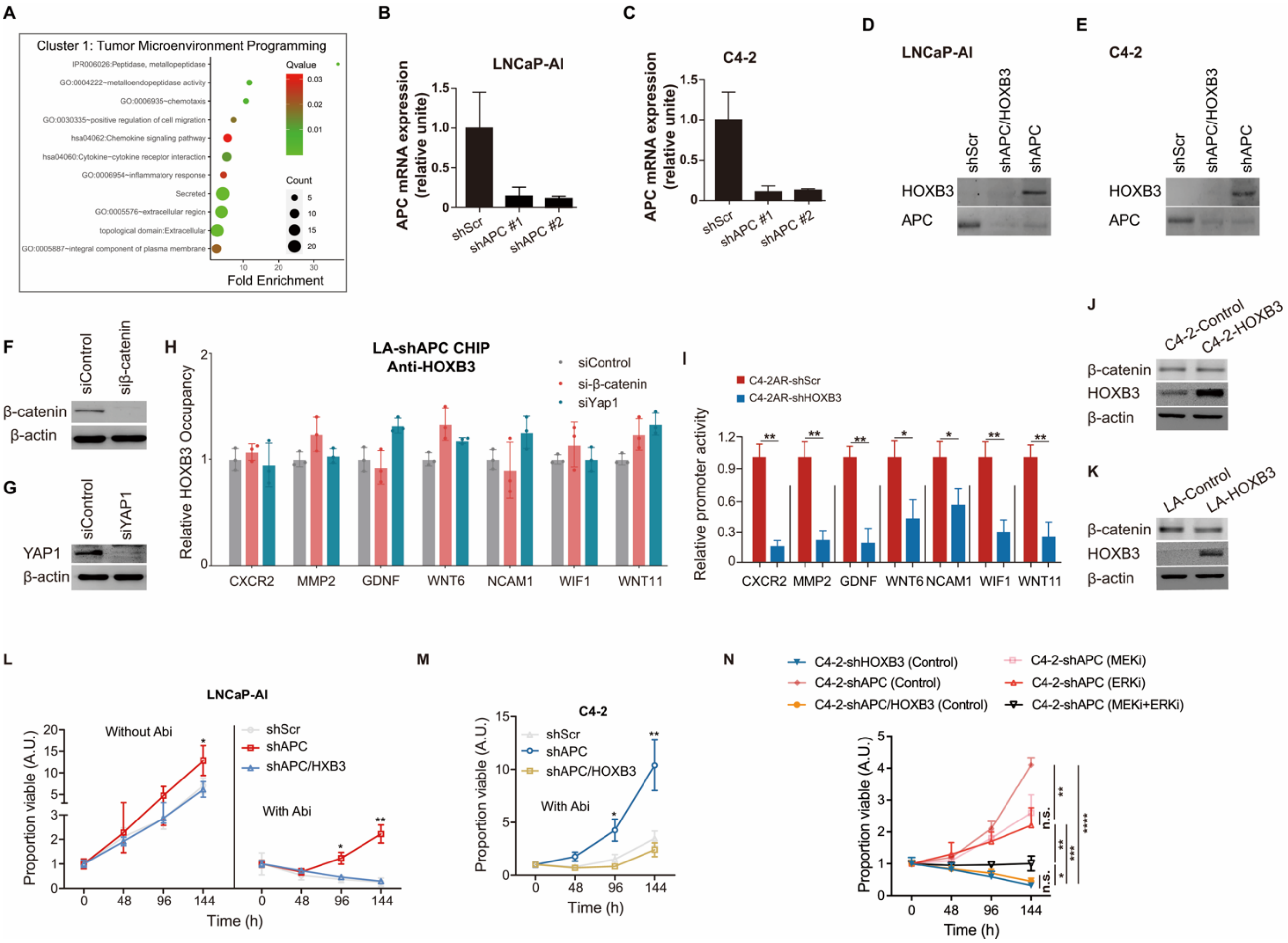


**Figure S4.** HOXB3 is an effector of APC-defect driven resistance to novel hormonal therapy in CRPC, related to Figures 6. (**A**) Overlapped genes showing significant enrichments in Tumor Microenvironment Programming determined by Functional Annotation Tool, including Metallopeptidase, Chemotaxis and Chemokine signaling pathway. (**B and C**) APC knockdown in LNCaP-AI or C4-2 cells. APC mRNA was measured by qRT-PCR in LNCaP-AI or C4-2 cells infected by shScr or shAPC. (**D and E**) APC and HOXB3 double knockdown in LNCaP-AI or C4-2 cells. Western-blot of APC and HOXB3 in LNCaP-AI or C4-2 cells infected by shHOXB3, sh-APC or both (shHOXB3/APC). (**F and G**) Western blots of β-catenin or Yap1 in LA-shAPC cells transfected with siRNA targeted β-catenin or Yap1. (**H**) qRT-PCRs assay based on CHIP experiments showing HOXB3 binds to the promoters of representative WNT-regulated genes. ChIP experiments using anti-HOXB3 antibody were performed in LA-shAPC-siβ-catenin and LA-shAPC-siYap1 cells. (**I**) Luciferase assays for promoter activities of representative WNT-regulated genes in C4-2AR-shScr and C4-2AR-shHOXB3 24h post

transfection. (**J** and **K**) Western blot of β-catenin in LA-HOXB3 vs. LA-Control and C4-2-HOXB3 vs. C4-2-Control. (**L**) MTT assays in LNCaP-AI cells infected by shHOXB3, sh-APC or shHOXB3+shAPC (shAPC/HOXB3). All of these cells were cultured in 10% CSS-FBS medium with or without abiraterone. (**M**) MTT assays in C4-2 cells infected by shHOXB3, sh-APC or shHOXB3/APC. The cells were cultured in medium with abiraterone. (**N**) MTT assays in C4-2 cells infected by shHOXB3, sh-APC or shHOXB3+shAPC (shAPC/HOXB3). All of these cells were cultured in 10% CSS-FBS medium with abiraterone. And with or without specific inhibitors of MEK1/2, ERK1/2. Error bars indicate mean ± SD. *represents $p < 0.05$, **represents $p < 0.01$.

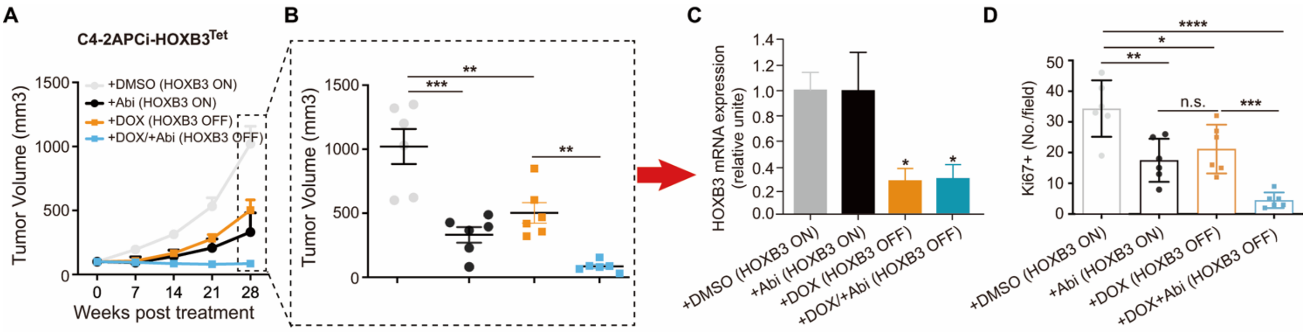


**Figure S5.** HOXB3 suppression sensitizes APC-defective CRPC xenografts to abiraterone In vivo, related to Figure 7.

(**A** and **B**) Caliper measurements were taken weekly for C4-2 xenografts. n = 6 mice per group. (A) Line graph with bar showing changes of tumor volumes weekly in each group; (B) Scatter plots with bar showing individual tumor volumes at 28 days post-randomization in each group. (**C**) Bar graph showing HOXB3 stable suppression in HOXB3-OFF C4-2 xenografts at 28 days post-randomization. HOXB3 mRNA levels were measured by qRT-PCR. (**D**) Scatter plots with bar showing quantified results of immunofluorescent staining of Ki67 in C4-2 xenografts. Error bars indicate mean ± SD. *represents $p < 0.05$, **represents $p < 0.01$.

Table S5 Primers were from primer bank or designed using primer 5

| For RT-qPCR | | |
|---|---|---|
| | Forward Sequence | Reverse Sequence |
| WNT3A | GCCCGTGCTGGACAAAGCT | TTCTGCACATGAGCGTGTCACT |
| HOXB3 | GGCAAACGTCCAAGCTGAA | CTCCAGCTCCACCAGCTGCG |
| CCND1 | TCAAATGTGTGCAGAAGGAGGT | GACAGGAAGCGGTCCAGGTA |
| CDCA3 | TGGTATTGCACGGACACCTA | TGTTTCACCAGTGGGCTTG |
| APC | GCCCCTGACCAAAAAGGAAC | TGGCAGCAACAGTCCCACTA |
| GAPDH | GAAATCCCATCACCATCTTCCAGG | GAGCCCCAGCCTTCTCCATG |
| MMP2 | GATACCCCTTTGACGGTAAGGA | CCTTCTCCCAAGGTCCATAGC |
| CXCR2 | CCTGTCTTACTTTTCCGAAGGAC | TTGCTGTATTGTTGCCCATGT |
| GDNF | GGCAGTGCTTCCTAGAAGAGA | AAGACACAACCCCGGTTTTTG |
| NCAM1 | GGCATTTACAAGTGTGTGGTTAC | TTGGCGCATTCTTGAACATGA |
| WNT6 | GGCAGCCCCTTGGTTATGG | CTCAGCCTGGCACAACTCG |
| WNT11 | GGAGTCGGCCTTCGTGTATG | GCCCGTAGCTGAGGTTGTC |
| WIF1 | TCTCCAAACACCTCAAAATGCT | GACACTCGCAGATGCGTCT |
| For CHIP | | |
| | Forward Sequence | Reverse Sequence |
| MMP2 | AGACGGTTGTCACAGGGAG | ATGGCAATGTGGGGAGGT |
| CXCR2 | GCATACAGTTTCAGGGAAAGAG | CGCTAGCTATTGATGCAGTCT |
| GDNF | AGCATGAAAATGGAGCCTAGGA | GCTGAAGTCAGAGGCTTTGCA |

| | | |
|---|---|---|
| NCAM1 | GGAGACTGCGTGTGAAAGAGC | AACTGGGATGGGAGCGAGGAG |
| WNT6 | AACCCCGCAGAGGCTAGGAGA | GGGGTGGCAGTTGCGACAGT |
| WNT11 | CGAATTGCCCCAGCTTACTG | TGGGGCTTATCCCTGCAATA |
| WIF1 | TCTGGAGCATCCTACCTTGC | ATGAGCACTCTAGCCTGATGG |
| GAPDH | TTGACTCACCCTGCCCTCAATATC | TTTCATTCCATCCAGCCTGGGG |
