# Supplementary material for: HOXB3 drives WNT-activation associated progression in castration-resistant prostate cancer
Source: Cell Death Dis. 2023 Mar 27;14(3):215. doi: 10.1038/s41419-023-05742-y (PMC10042887; doi:10.1038/s41419-023-05742-y)
Supplement: Supplementary file 5 — Checklist [file 41419_2023_5742_MOESM5_ESM.pdf]

SPRINGER NATURE

Corresponding Author Name: Zhiqun Shang
Manuscript Number: CDDIS-22-3048R

Reporting Summary

Springer Nature wishes to improve the reproducibility of the work that we publish. This checklist is used to ensure good reporting standards and to improve the reproducibility. Please respond completely to all questions relevant to your manuscript. For more information, please read the journal's Guide to Authors.

☑ **Check here to confirm that the following information is available in the Material & Methods section:**

- **The exact sample size (*n*)** for each experimental group/condition, given as a number, not a range
- **A description of the sample collection** allowing the reader to understand whether the samples represent technical or biological replicates (including how many animals, litters, culture, etc.)
- **A statement of how many times the experiment shown was replicated in the laboratory**
- **Definitions of statistical methods and measures**: For small sample sizes (n<5) descriptive statistics are not appropriate, instead plot individual data points
    - Very common tests, such as *t*-test, simple χ^2 tests, Wilcoxon and Mann-Whitney tests, can be unambiguously identified by name only, but more complex techniques should be described in the methods section
    - Are tests one-sided or two-sided?
    - Are there adjustments for multiple comparisons?
    - **Statistical test results**, e.g., ***P* values**
    - Definition of **'center values'** as **median or mean**;
    - Definition of **error bars as s.d. or s.e.m. or c.i.**

Please ensure that the answers to the following questions are reported in the manuscript itself. We encourage you to include a specific subsection in the methods section for statistics, reagents and animal models. Below, provide the page number or section and paragraph number.

Statistics and general methods

Question	Reported in section/paragraph or page #
1. How was the sample size chosen to ensure adequate power to detect a pre-specified effect size? (Give section/paragraph or page #)	Reported in "Methods-Statistical analysis" section (page # 15/41). No statistical methods were used to predetermine the sample size.
For animal studies, include a statement about sample size estimate even if no statistical methods were used.	Reported in "Methods-Murine prostate tumor xenograft model" section (page #14/41). No statistical methods were used to predetermine the sample size.
2. Describe inclusion/exclusion criteria if samples or animals were excluded from the analysis. Were the criteria pre-established? (Give section/paragraph or page #)	The criteria were pre-established, including the age of mice and the size of tumors. See "Methods-Murine prostate tumor xenograft model" section (page #14/41).
3. If a method of randomization was used to determine how samples/animals were allocated to experimental groups and processed, describe it. (Give section/paragraph or page #)	Nude mice were randomly allocated to experimental groups. See "Methods-Murine prostate tumor xenograft model" section (page #14/41).
For animal studies, include a statement about randomization even if no randomization was used.	Animals are randomized based on the body weight.

4. If the investigator was blinded to the group allocation during the experiment and/or when assessing the outcome, state the extent of blinding. (Give section/paragraph or page #)	Reported in "Methods-Murine prostate tumor xenograft model" section (page #14/41). The investigator measured tumors blinding to group allocation during experiments.
For animal studies, include a statement about blinding even if no blinding was done.	The investigators measured tumors blinding to group allocation during experiments.
5. For every figure, are statistical tests justified as appropriate?	The statistical test used in each figure is indicated in the figure legends.
Do the data meet the assumptions of the tests (e.g., normal distribution)?	Given that sample size is less than 30, we assume that the data did not follow Gaussian distribution and therefore [illegible]
Is there an estimate of variation within each group of data?	Yes, Standard deviation was included.
Is the variance similar between the groups that are being statistically compared? (Give section/paragraph or page #)	page #14/41

Reagents

Reported in section/paragraph or page #

6. Report the source of antibodies (vendor and catalog number)	Yes, it is reported in the "Methods" section.
7. Identify the source of cell lines and report if they were recently authenticated (e.g., by STR profiling) and tested for mycoplasma contamination	Yes, the source of the cell lines are mentioned in the Methods section of the manuscript. We test for mycoplasma contamination every six months at the laboratory.

Animal Models

Reported in section/paragraph or page #

8. Report species, strain, sex and age of animals	Reported in "Methods-Murine prostate tumor xenograft [illegible]
9. For experiments involving live vertebrates, include a statement of compliance with ethical regulations and identify the committee(s) approving the experiments.	Reported in "Methods-Murine prostate tumor xenograft model" section (page #14/41).

10. We recommend consulting the ARRIVE guidelines (*PLoS Biol.* **8**(6), e1000412,2010) to ensure that other relevant aspects of animal studies are adequately reported.

Human subjects

	Reported in section/paragraph or page #
11. Identify the committee(s) approving the study protocol.	Reported in "Methods-Patients" section (page #6/41). Tissue specimens were obtained from a Prospective study [illegible]
12. Include a statement confirming that informed consent was obtained from all subjects.	Yes, It's reported in "Methods-Patients" section (page #6/41).
13. For publication of patient photos, include a statement confirming that consent to publish was obtained.	Not applicable
14. Report the clinical trial registration number (at ClinicalTrials.gov or equivalent).	Reported in "Methods-Patients" section (page #6/41): NCT03176381.

15. For phase II and III randomized controlled trials, please refer to the CONSORT statement and submit the CONSORT checklist with your submission.

16. For tumor marker prognostic studies, we recommend that you follow the REMARK reporting guidelines.

Data deposition

	Reported in section/paragraph or page #
17. Provide accession codes for deposited data. Data deposition in a public repository is mandatory for:<br>a. Protein, DNA and RNA sequences<br>b. Macromolecular structures<br>c. Crystallographic data for small molecules<br>d. Microarray data	The gene expression data for DHT-treated LNCaP cells generated in this study has been submitted to the NCBI Gene Expression Omnibus under the accession number GSE163539. The gene expression data and binding data in this study have been deposited into CNGB Sequence Archive (CNSA) of China National GeneBank DataBase (CNGBdb) with accession number CNP0001560 and CNP0001628.

Deposition is strongly recommended for many other datasets for which structured public repositories exist; more details on our data policy are available in the Guide to Authors. We encourage the provision of other source data in supplementary information or in unstructured repositories such as Figshare and Dryad. We encourage publication of Data Descriptors (see Scientific Data) to maximize data reuse.

18. If computer code was used to generate results that are central to the paper's conclusions, include a statement in the Methods section under "**Code availability**" to indicate whether and how the code can be accessed. Include version information as necessary and any restrictions on availability.	Yes, See "RNA-seq and pathway analysis" section in the Material and Method of the manuscript.
