# Supplementary material for: HOXB3 drives WNT-activation associated progression in castration-resistant prostate cancer
Source: Cell Death Dis. 2023 Mar 27;14(3):215. doi: 10.1038/s41419-023-05742-y (PMC10042887; doi:10.1038/s41419-023-05742-y)

Figure 3F

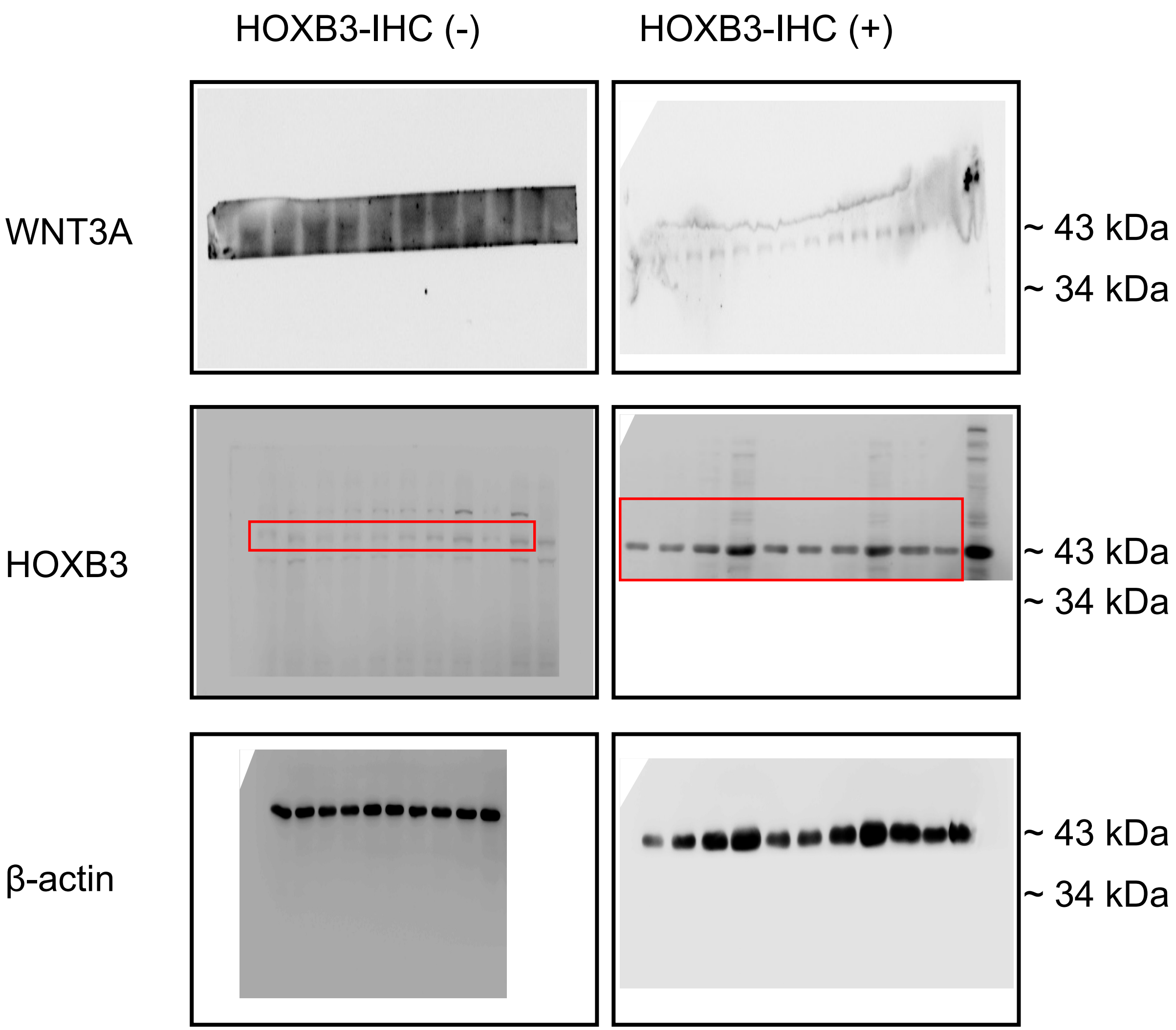

HOXB3-IHC (-)
HOXB3-IHC (+)
WNT3A
~ 43 kDa
~ 34 kDa
HOXB3
~ 43 kDa
~ 34 kDa
β-actin
~ 43 kDa
~ 34 kDa

Figure 4A

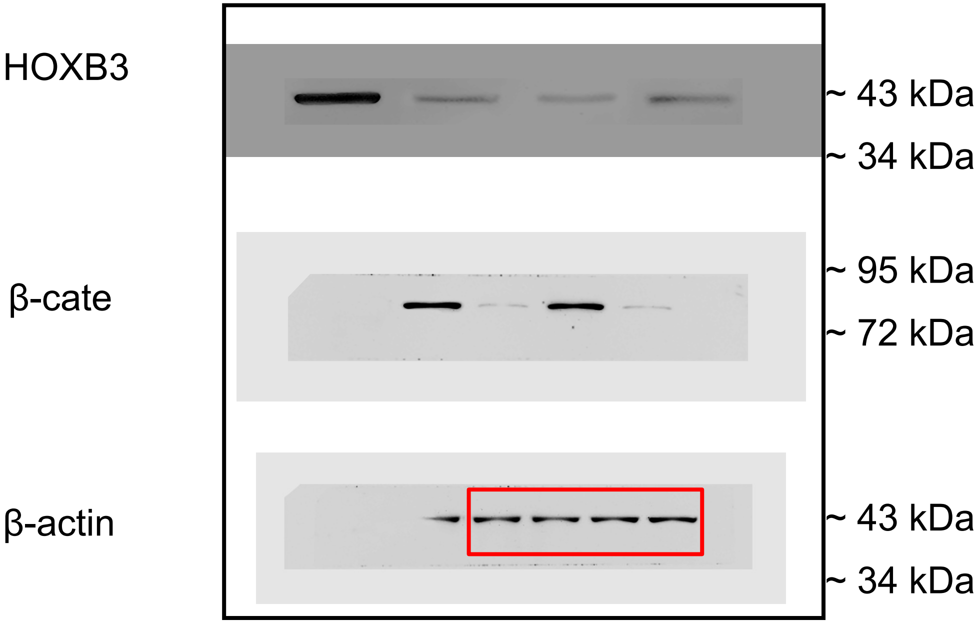

Figure 4B

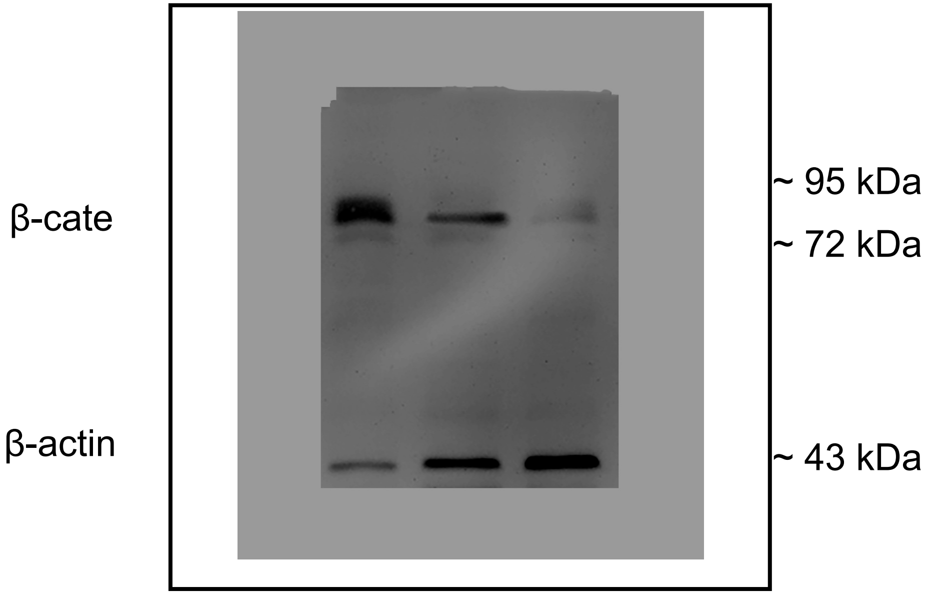

Figure 4C

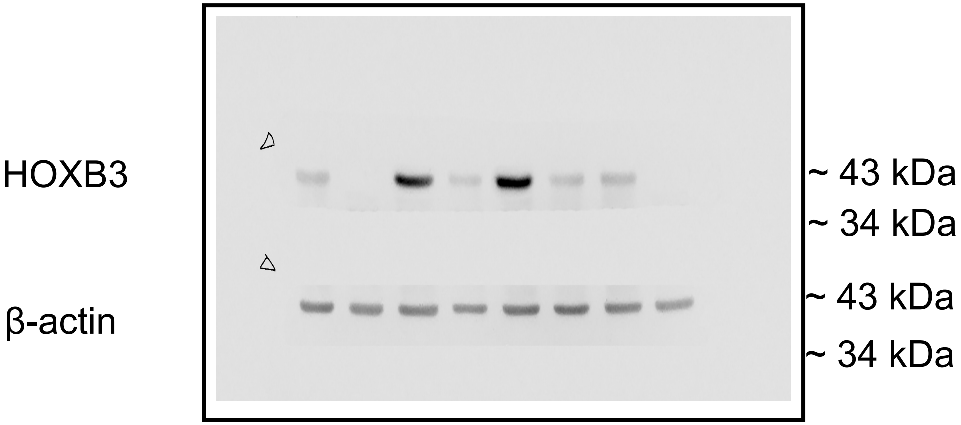

Figure 4D

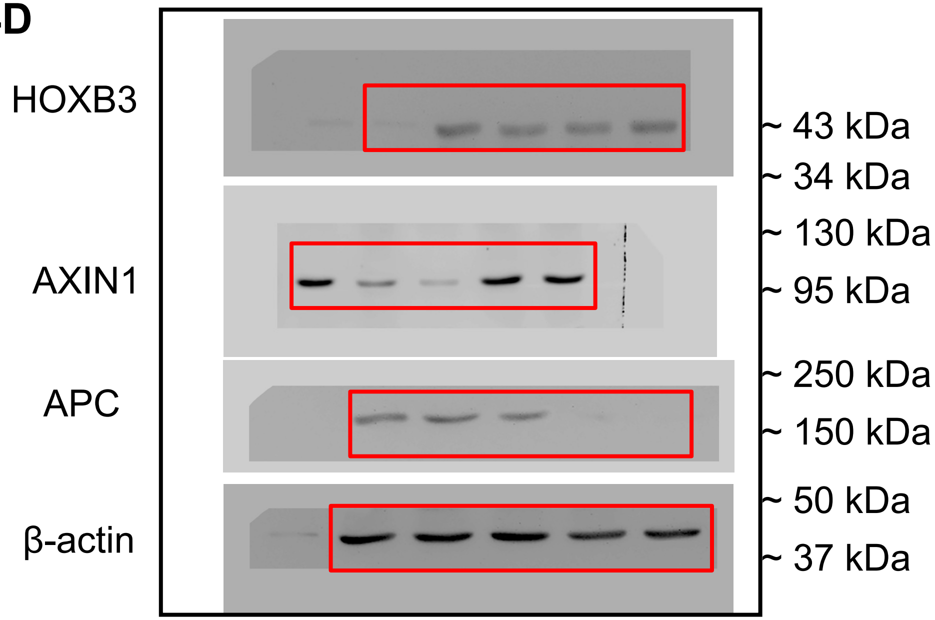

Figure 5A

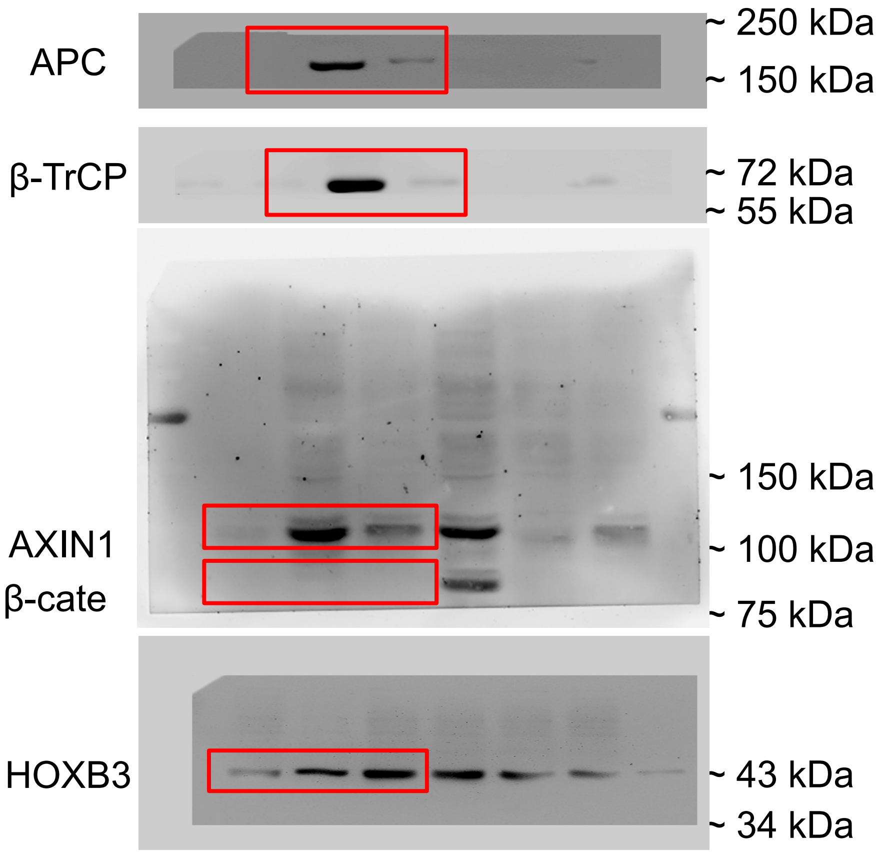

Figure 5C

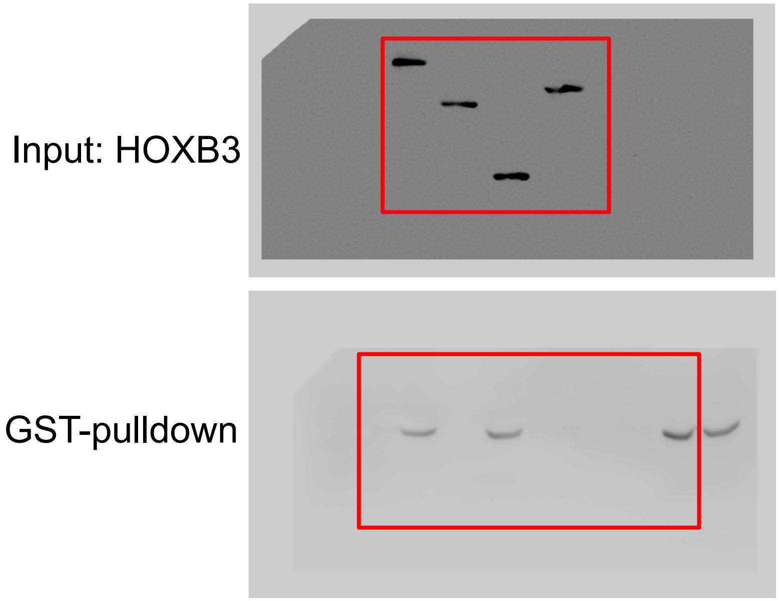

Figure 5D

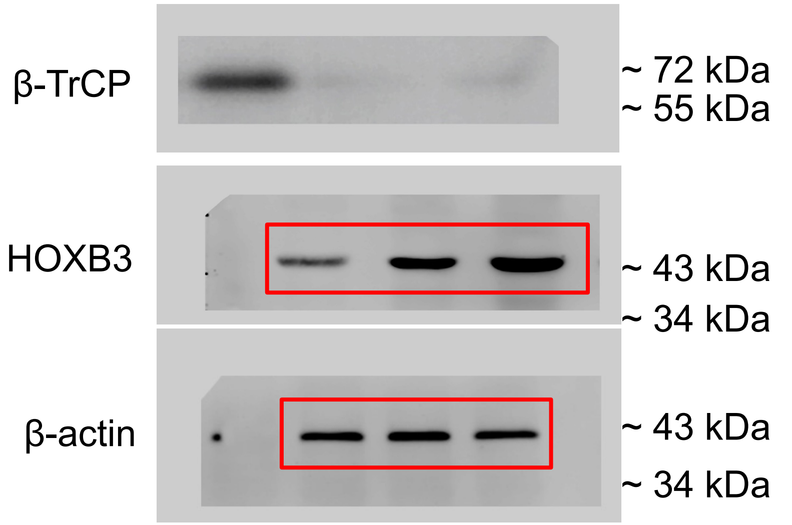

Figure 5E

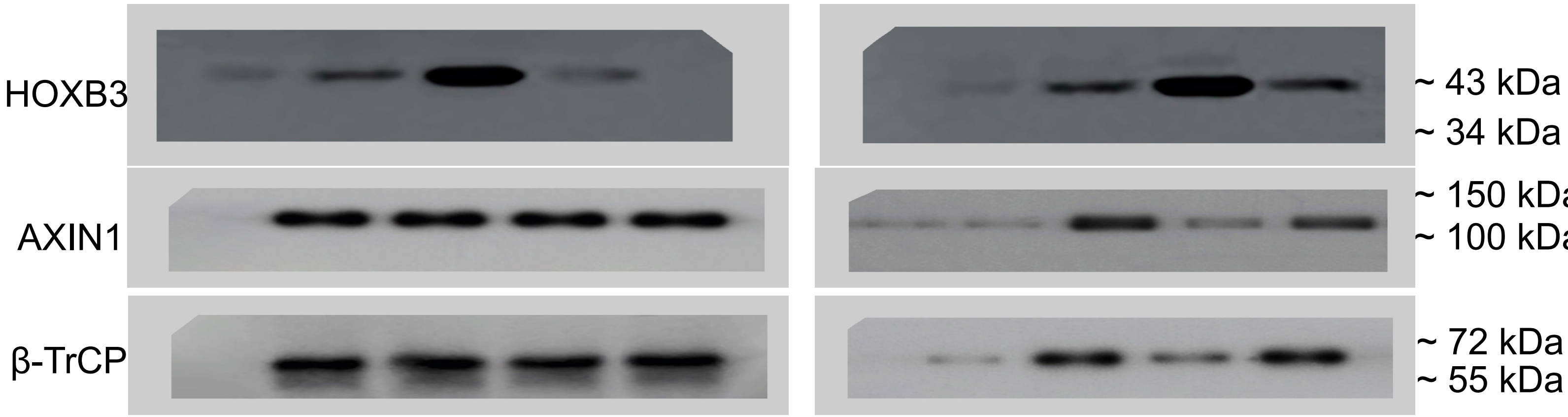

Figure 5F

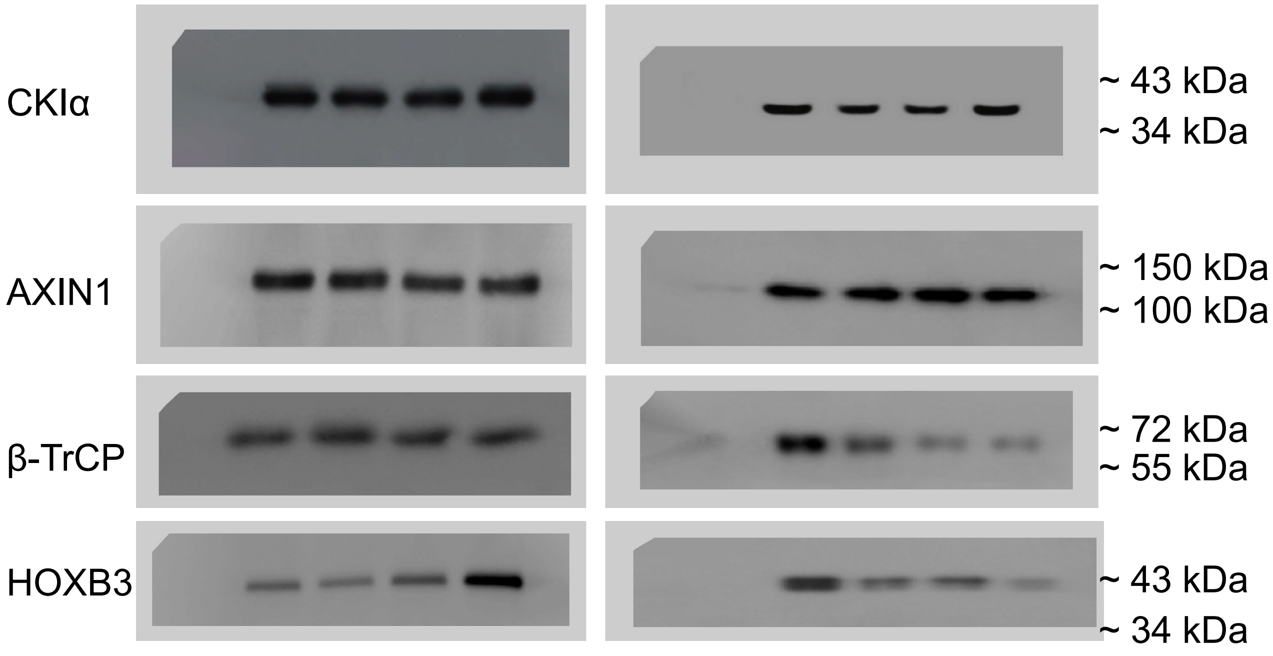

Figure 5H

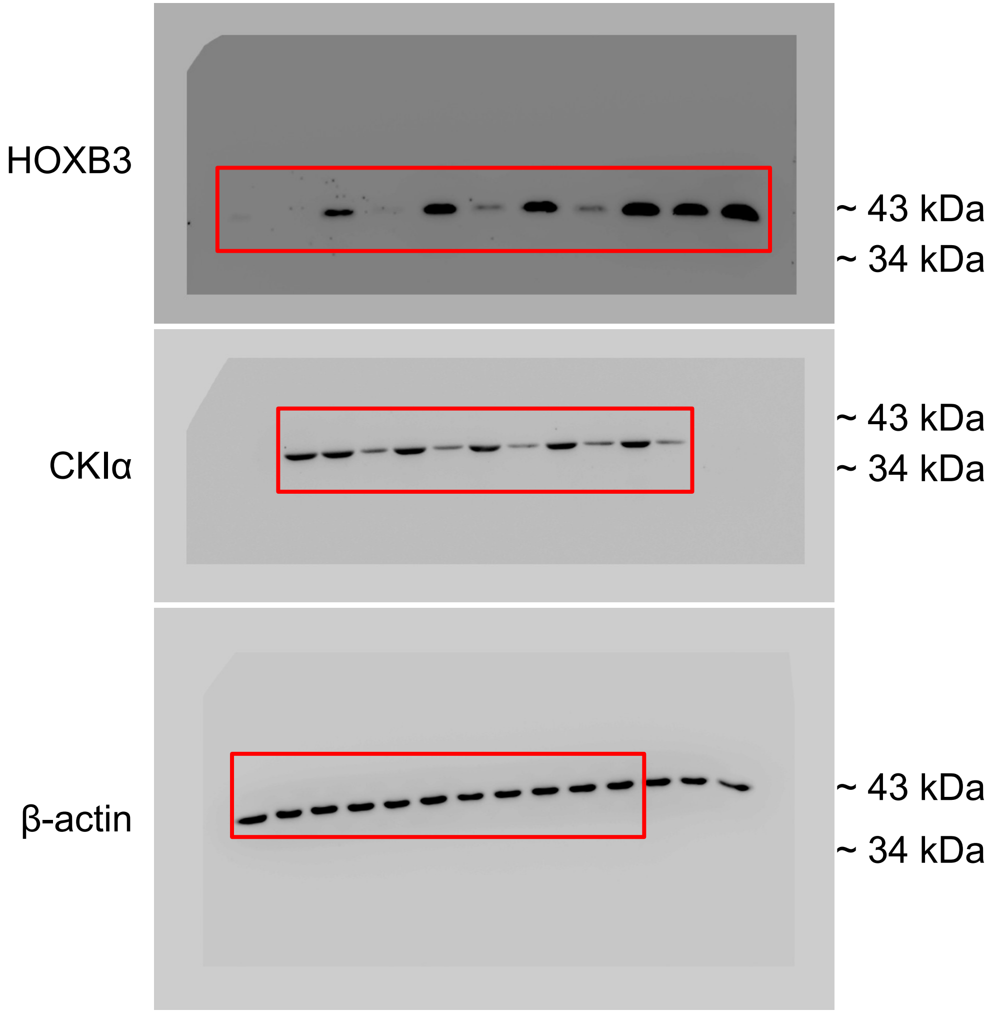

Figure 6E

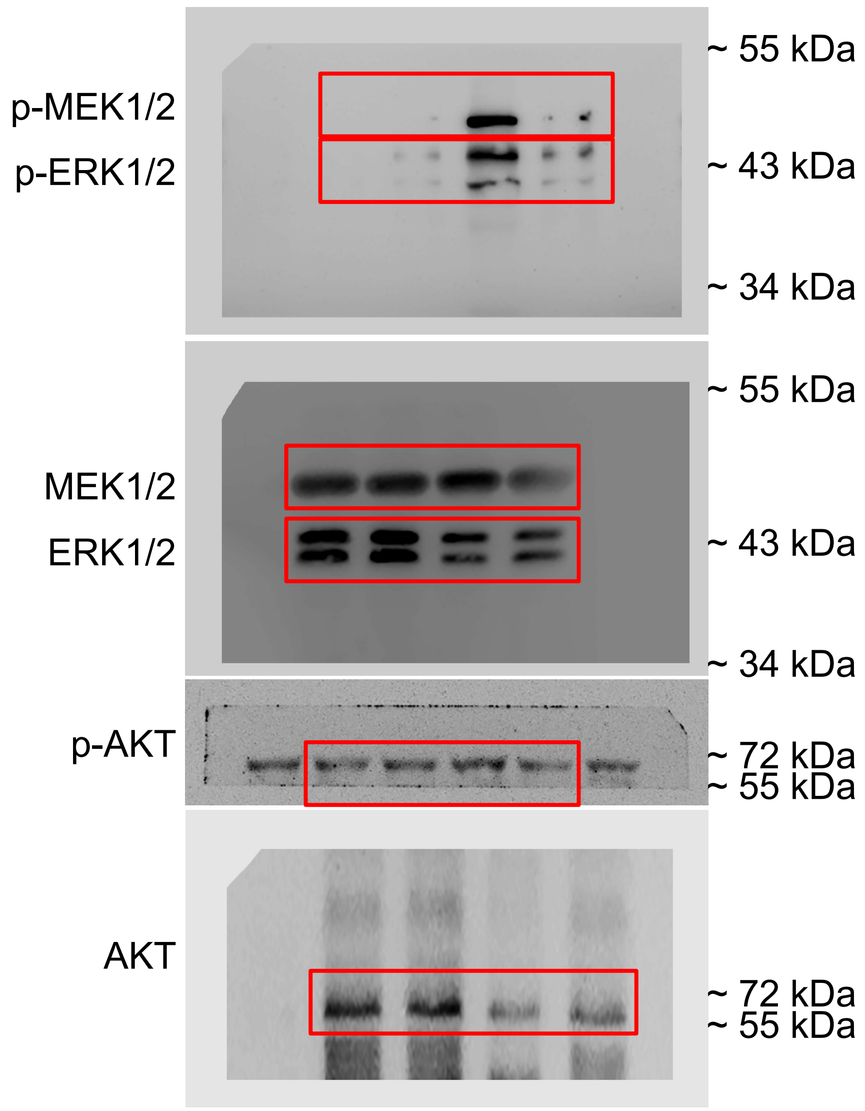
p-MEK1/2
p-ERK1/2
~ 55 kDa
~ 43 kDa
~ 34 kDa
MEK1/2
ERK1/2
~ 55 kDa
~ 43 kDa
~ 34 kDa
p-AKT
~ 72 kDa
~ 55 kDa
AKT
~ 72 kDa
~ 55 kDa

Figure S1A

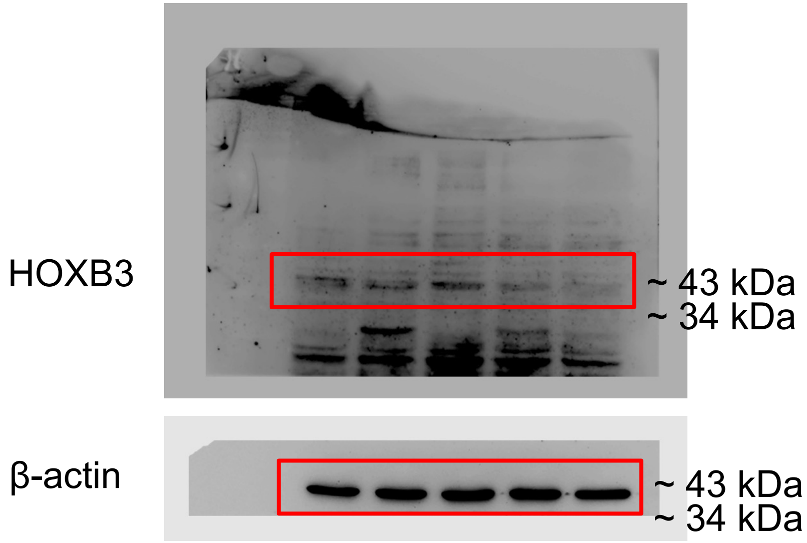

Figure S1B-C

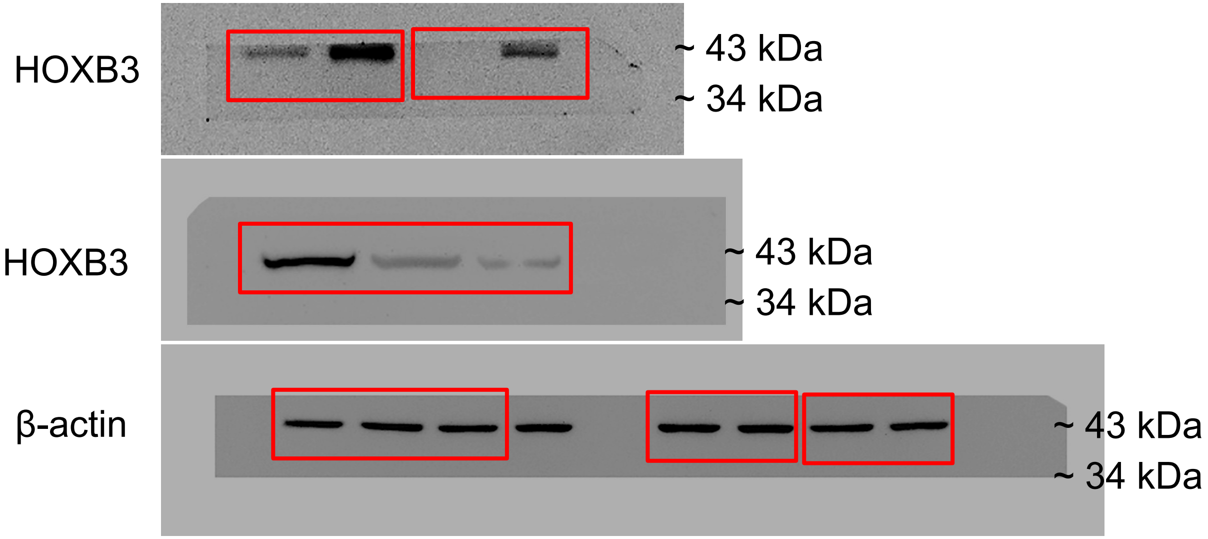

Figure S3A

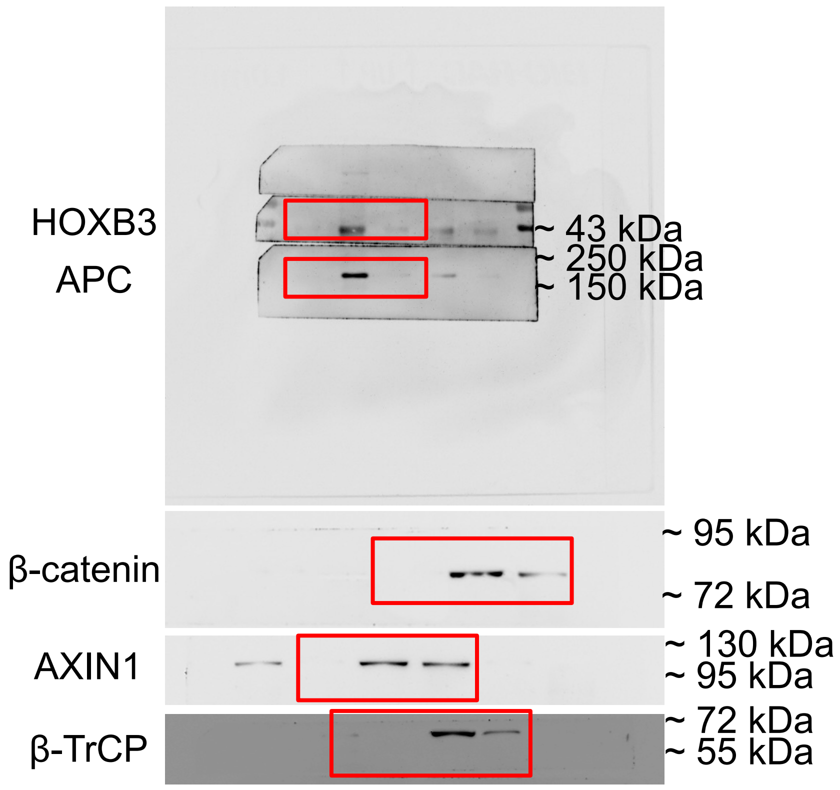

Figure S3B

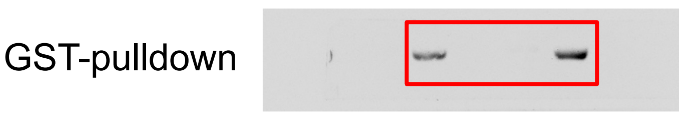

Figure S3C

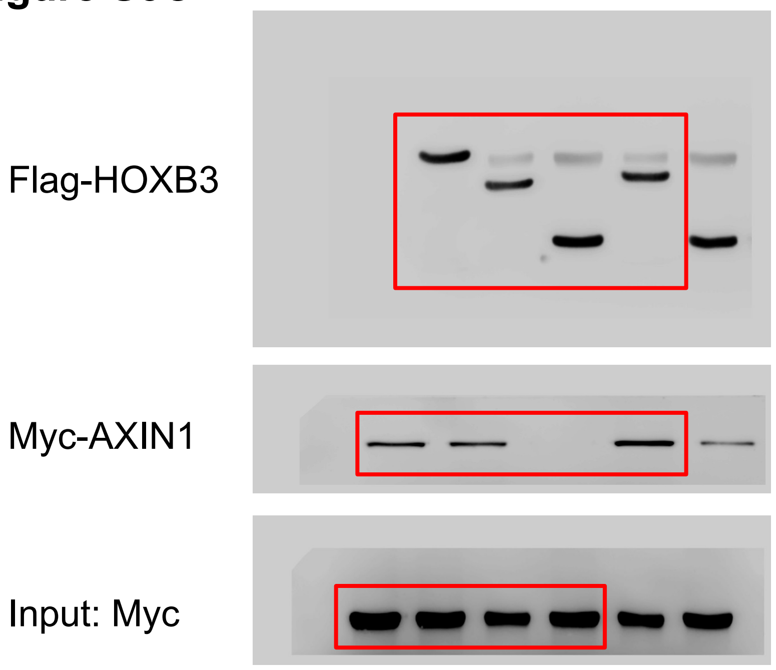

Figure S3F

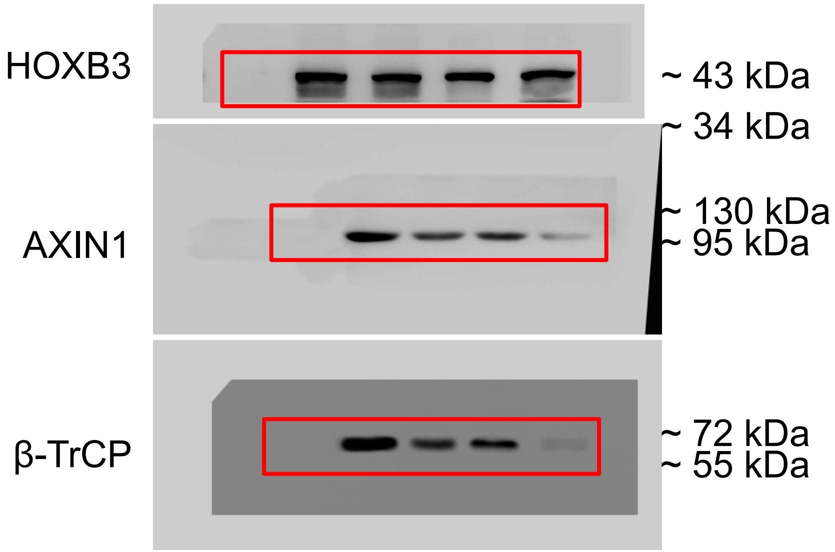

Figure S4D & S4E

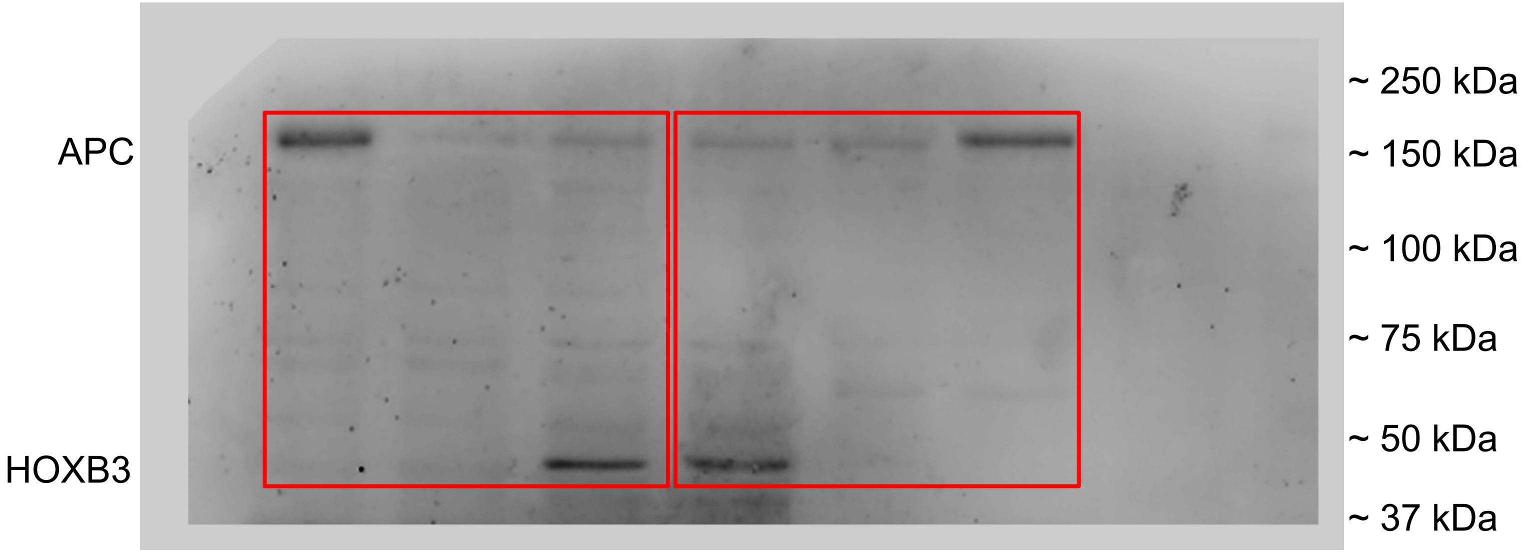

Figure S4F & S4G

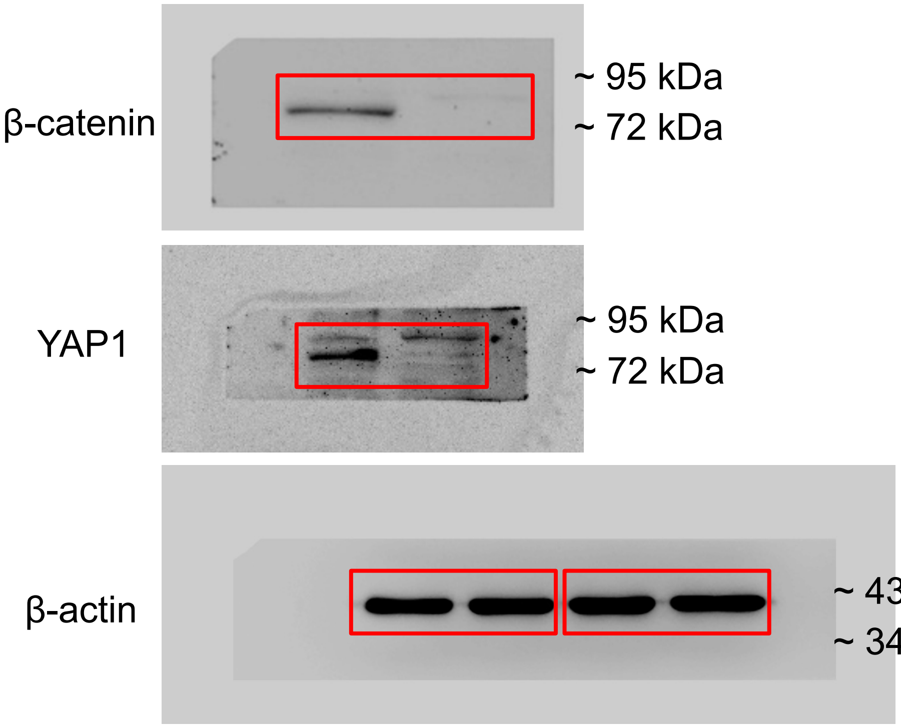

Figure S4H & S4I

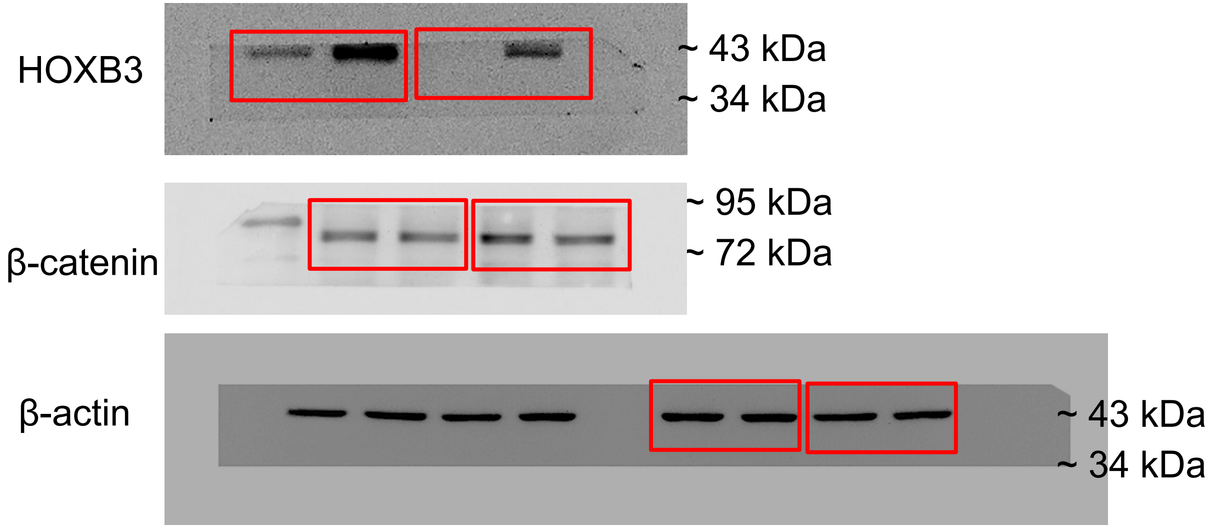

Supplement: Supplementary file 7 — Original Data File [file 41419_2023_5742_MOESM7_ESM.pdf]
