# Supplementary material for: HOXB3 drives WNT-activation associated progression in castration-resistant prostate cancer
Source: Cell Death Dis. 2023 Mar 27;14(3):215. doi: 10.1038/s41419-023-05742-y (PMC10042887; doi:10.1038/s41419-023-05742-y)
Supplement: Supplementary file 6 — Authorship Confirmation [file 41419_2023_5742_MOESM6_ESM.pdf]

Re:Authorship Confirmation

ToMe

S Shimiao 2023-03-12 10:48
收件人：我 详情

Dear Prof. Shang,

I agree with this authorship.

Shimiao Zhu

发件人："尚芝群" <zhiqun_shang@tmu.edu.cn>
发送日期：2023-03-12 10:35:59
收件人：Shimiao <zhushimiao@tmu.edu.cn>
主题：Authorship Confirmation

隐藏引用内容

Dear Dr. Zhu,

As determined after discusion, the authorship has been updated as listed in the figure at bottom. Before publication, agreements from all co-authors should be suggestion.
Thank you!

Shimiao Zhu[1], Zhao Yang[1], Zheng Zhang[1], Hongli Zhang[1], Songyang Li[1], Tao Wu[2], Xuanrong Chen[1], Jianing Guo[3], Aixiang Wang[4], Hao Tian[1], Jianpeng Yu[1], Changwen Zhang[1], Lei Su[5], Zhiqun Shang[1,**] , Changyi Quan[1,*], Yuanjie Niu[1,***]

[1]Department of Urology, Tianjin Institute of Urology, The Second Hospital of Tianjin Meidical University, Tianjin, 300211, China. [2]Department of Urology, Shenzhen Hospital, Southern Medical University, Shenzhen, China. [3]Department of Pathology, The Second Hospital of Tianjin Medical University, Tianjin, China. [4]Institute of Urology, Peking University; National Urological Cancer Center; Beijing, China. [5]Department of Urology, People's Hospital of Rizhao, Rizhao, Shandong, China.

[*]Corresponding author. Tel: +86 022 88328607; E-mail: quancahngyi@tmu.edu.cn; ORCID: 0000-0002-3511-5604

[**]Corresponding author. Tel: +86 022 88329296; E-mail: zhiqun_shang@tmu.edu.cn; ORCID: 0000-0001-8263-707X

[***]Corresponding author. Tel: +86 022 88329296; E-mail: niuyuanjie9317@163.com; ORCID: 0000-0002-9266-727X

Shimiao Zhu, Zhao Yang, Zheng Zhang and Hongli Zhang contributed equally to this work.

Zhiqun Shang PhD&MD
Professor of Tianjin Institute of Urology
The Second Hospital of Tianjin Medical University
TEL:18722292731

Re:Authorship Confirmation

ToMe

Z Zhao 2023-03-12 15:33
收件人：我 详情

Dear Prof. Shang,

I agree with this authorship.

Zhao Yang

发件人："尚芝群" <zhiqun_shang@tmu.edu.cn>
发送日期：2023-03-12 10:35:59
收件人：YangZ <YangZhao77@tmu.edu.cn>
主题：Authorship Confirmation

隐藏引用内容

Dear Dr. Yang,

As determined after discussion, the authorship has been updated as listed in the figure at bottom. Before publication, agreements from all co-authors should be suggestion.
Thank you!

Shimiao Zhu[1], Zhao Yang[1], Zheng Zhang[1], Hongli Zhang[1], Songyang Li[1], Tao Wu[2], Xuanrong Chen[1], Jianing Guo[3], Aixiang Wang[4], Hao Tian[1], Jianpeng Yu[1], Changwen Zhang[1], Lei Su[5], Zhiqun Shang[1,**] , Changyi Quan[1,*], Yuanjie Niu[1,***]

[1]Department of Urology, Tianjin Institute of Urology, The Second Hospital of Tianjin Meidical University, Tianjin, 300211, China. [2]Department of Urology, Shenzhen Hospital, Southern Medical University, Shenzhen, China. [3]Department of Pathology, The Second Hospital of Tianjin Medical University, Tianjin, China. [4]Institute of Urology, Peking University; National Urological Cancer Center; Beijing, China. [5]Department of Urology, People's Hospital of Rizhao, Rizhao, Shandong, China.

*Corresponding author. Tel: +86 022 88328607; E-mail: quancahngyi@tmu.edu.cn; ORCID: 0000-0002-3511-5604

**Corresponding author. Tel: +86 022 88329296; E-mail: zhiqun_shang@tmu.edu.cn; ORCID: 0000-0001-8263-707X

***Corresponding author. Tel: +86 022 88329296; E-mail: niuyuanjie9317@163.com; ORCID: 0000-0002-9266-727X

Shimiao Zhu, Zhao Yang, Zheng Zhang and Hongli Zhang contributed equally to this work.

Zhiqun Shang PhD&MD
Professor of Tianjin Institute of Urology
The Second Hospital of Tianjin Medical University
TEL:18722292731

Re:Authorship Confirmation

ToMe

z 张正 2023-03-12 11:22
收件人：我 详情

Dear Prof. Shang,

I agree with this authorship.

Zheng Zhang

发件人："尚芝群" <zhiqun_shang@tmu.edu.cn>
发送日期：2023-03-12 10:35:59
收件人：张正 <494595402@qq.com>
主题：Authorship Confirmation

隐藏引用内容

Dear Dr. Zhang,

As determined after discussion, the authorship has been updated as listed in the figure at bottom. Before publication, agreements from all co-authors should be suggestion.
Thank you!

Shimiao Zhu[1], Zhao Yang[1], Zheng Zhang[1], Hongli Zhang[1], Songyang Li[1], Tao Wu[2], Xuanrong Chen[1], Jianing Guo[3], Aixiang Wang[4], Hao Tian[1], Jianpeng Yu[1], Changwen Zhang[1], Lei Su[5], Zhiqun Shang[1,**], Changyi Quan[1,*], Yuanjie Niu[1,***]

[1]Department of Urology, Tianjin Institute of Urology, The Second Hospital of Tianjin Meidical University, Tianjin, 300211, China. [2]Department of Urology, Shenzhen Hospital, Southern Medical University, Shenzhen, China. [3]Department of Pathology, The Second Hospital of Tianjin Medical University, Tianjin, China. [4]Institute of Urology, Peking University; National Urological Cancer Center; Beijing, China. [5]Department of Urology, People's Hospital of Rizhao, Rizhao, Shandong, China.

[*]Corresponding author. Tel: +86 022 88328607; E-mail: quancahngyi@tmu.edu.cn; ORCID: 0000-0002-3511-5604

[**]Corresponding author. Tel: +86 022 88329296; E-mail: zhiqun_shang@tmu.edu.cn; ORCID: 0000-0001-8263-707X

[***]Corresponding author. Tel: +86 022 88329296; E-mail: niuyuanjie9317@163.com; ORCID: 0000-0002-9266-727X

Shimiao Zhu, Zhao Yang, Zheng Zhang and Hongli Zhang contributed equally to this work.

Zhiqun Shang PhD&MD
Professor of Tianjin Institute of Urology
The Second Hospital of Tianjin Medical University
TEL:18722292731

快捷回复全部

天津醫科大學 TIANJIN MEDICAL UNIVERSITY z zhiqun_shang@tmu.edu.cn 设置 下载 帮助与反馈 English 退出登录 回到旧版

收件箱 Authorship ... 系统退信 Fw:CDDIS-2... 无主题 Authorship ... Track the sta... Re:Authorsh...

邮箱 12 日历 通讯录 消息 云文档

写邮件

搜索邮件

- 收件箱
- 未读邮件 28
- 发件箱
- 红旗邮件
- 任务邮件
- 稍后处理
- 草稿箱 11
- 垃圾邮件
- 已删除
- 广告邮件
- 订阅邮件

新建文件夹

自定义专属文件夹，高效管理各类邮件

新建文件夹

标签 +

< 返回 回复 回复全部 转发 删除 标记为 移动 举报 分享 更多

Re:Authorship Confirmation

ToMe

z 张弘历 2023-03-12 15:35
收件人：我 详情

Dear Prof. Shang,

I agree this authorship.

Hongli Zhang

发件人："尚芝群"
<zhiqun_shang@tmu.edu.cn>发送日期：
2023-03-12 10:35:59
收件人：张弘历 <zhl158182@tmu.edu.cn>
主题：Authorship Confirmation
隐藏引用内容

Dear Dr. Zhang,

As determined after discussion, the authorship has been updated as listed in the figure at bottom. Before publication, agreements from all co-authors should be suggestion.
Thank you!

Shimiao Zhu[1], Zhao Yang[1], Zheng Zhang[1], Hongli Zhang[1], Songyang Li[1], Tao Wu[2], Xuanrong Chen[1], Jianing Guo[3], Aixiang Wang[4], Hao Tian[1], Jianpeng Yu[1], Changwen Zhang[1], Lei Su[5], Zhiqun Shang[1,**] , Changyi Quan[1,*], Yuanjie Niu[1,***]

[1]Department of Urology, Tianjin Institute of Urology, The Second Hospital of Tianjin Meidical University, Tianjin, 300211, China. [2]Department of Urology, Shenzhen Hospital, Southern Medical University, Shenzhen, China. [3]Department of Pathology, The Second Hospital of Tianjin Medical University, Tianjin, China. [4]Institute of Urology, Peking University; National Urological Cancer Center; Beijing, China. [5]Department of Urology, People's Hospital of Rizhao, Rizhao, Shandong, China.

[*]Corresponding author. Tel: +86 022 88328607; E-mail: quancahngyi@tmu.edu.cn; ORCID: 0000-0002-3511-5604

[**]Corresponding author. Tel: +86 022 88329296; E-mail: zhiqun_shang@tmu.edu.cn; ORCID: 0000-0001-8263-707X

[***]Corresponding author. Tel: +86 022 88329296; E-mail: niuyuanjie9317@163.com; ORCID: 0000-0002-9266-727X

Shimiao Zhu, Zhao Yang, Zheng Zhang and Hongli Zhang contributed equally to this work.

Zhiqun Shang PhD&MD
Professor of Tianjin Institute of Urology
The Second Hospital of Tianjin Medical University
TEL:18722292731

快捷回复全部

天津醫科大學 TIANJIN MEDICAL UNIVERSITY　z　zhiqun_shang@tmu.edu.cn　设置　下载　帮助与反馈　English　退出登录　回到旧版

收件箱　Authorship ...　系统退信　Fw:CDDIS-2...　无主题　Authorship ...　Track the sta...　Re:Authorsh...

邮箱　12 日历　通讯录　消息　云文档

写邮件

搜索邮件

- 收件箱
- 未读邮件 28
- 发件箱
- 红旗邮件
- 任务邮件
- 稍后处理
- 草稿箱 11
- 垃圾邮件
- 已删除
- 广告邮件
- 订阅邮件

新建文件夹

自定义专属文件夹，高效管理各类邮件

新建文件夹

标签 +

< 返回　回复　回复全部　转发　删除　标记为　移动　举报　分享　更多

Re:Authorship Confirmation

ToMe

S　Songyang　2023-03-12 16:40
收件人：我　详情

Dear Prof. Shang,

I agree with this authorship.

Songyang Li

发件人："尚芝群" <zhiqun_shang@tmu.edu.cn>
发送日期：2023-03-12 10:35:59
收件人：Songyang <2048590384@qq.com>
主题：Authorship Confirmation

隐藏引用内容

Dear Dr. Li,

As determined after discussion, the authorship has been updated as listed in the figure at bottom. Before publication, agreements from all co-authors should be suggestion.
Thank you!

Shimiao Zhu[1], Zhao Yang[1], Zheng Zhang[1], Hongli Zhang[1], Songyang Li[1], Tao Wu[2], Xuanrong Chen[1], Jianing Guo[3], Aixiang Wang[4], Hao Tian[1], Jianpeng Yu[1], Changwen Zhang[1], Lei Su[5], Zhiqun Shang[1,**] , Changyi Quan[1,*], Yuanjie Niu[1,***]

[1]Department of Urology, Tianjin Institute of Urology, The Second Hospital of Tianjin Meidical University, Tianjin, 300211, China. [2]Department of Urology, Shenzhen Hospital, Southern Medical University, Shenzhen, China. [3]Department of Pathology, The Second Hospital of Tianjin Medical University, Tianjin, China. [4]Institute of Urology, Peking University; National Urological Cancer Center; Beijing, China. [5]Department of Urology, People's Hospital of Rizhao, Rizhao, Shandong, China.

*Corresponding author. Tel: +86 022 88328607; E-mail: quancahngyi@tmu.edu.cn; ORCID: 0000-0002-3511-5604

**Corresponding author. Tel: +86 022 88329296; E-mail: zhiqun_shang@tmu.edu.cn; ORCID: 0000-0001-8263-707X

***Corresponding author. Tel: +86 022 88329296; E-mail: niuyuanjie9317@163.com; ORCID: 0000-0002-9266-727X

Shimiao Zhu, Zhao Yang, Zheng Zhang and Hongli Zhang contributed equally to this work.

Zhiqun Shang PhD&MD
Professor of Tianjin Institute of Urology
The Second Hospital of Tianjin Medical University
TEL:18722292731

快捷回复全部

天津醫科大學 TIANJIN MEDICAL UNIVERSITY z zhiqun_shang@tmu.edu.cn 设置 下载 帮助与反馈 English 退出登录 回到旧版
收件箱 Authorship ... 系统退信 Fw:CDDIS-2... 无主题 Authorship ... Track the sta... Re:Authorsh...
邮箱
写邮件
12
日历
搜索邮件
收件箱
未读邮件 28
通讯录
发件箱
消息
红旗邮件
云文档
任务邮件
稍后处理
草稿箱 11
垃圾邮件
已删除
广告邮件
订阅邮件
新建文件夹
自定义专属文件夹，高效管理各类邮件
新建文件夹
标签
返回 回复 回复全部 转发 删除 标记为 移动 举报 分享 更多

Re:Authorship Confirmation

ToMe

T Tao 2023-03-12 11:39
收件人：我 详情

Dear Prof. Shang,

I agree with this authorship.

Tao Wu

发件人："尚芝群" <zhiqun_shang@tmu.edu.cn>
发送日期：2023-03-12 10:35:59
收件人：Tao <Doctorwu1987@163.com>
主题：Authorship Confirmation

隐藏引用内容

Dear Dr. Wu,

As determined after discussion, the authorship has been updated as listed in the figure at bottom. Before publication, agreements from all co-authors should be suggestion.
Thank you!

Shimiao Zhu[1], Zhao Yang[1], Zheng Zhang[1], Hongli Zhang[1], Songyang Li[1], Tao Wu[2], Xuanrong Chen[1], Jianing Guo[3], Aixiang Wang[4], Hao Tian[1], Jianpeng Yu[1], Changwen Zhang[1], Lei Su[5], Zhiqun Shang[1,**] , Changyi Quan[1,*], Yuanjie Niu[1,***]

[1]Department of Urology, Tianjin Institute of Urology, The Second Hospital of Tianjin Meidical University, Tianjin, 300211, China. [2]Department of Urology, Shenzhen Hospital, Southern Medical University, Shenzhen, China. [3]Department of Pathology, The Second Hospital of Tianjin Medical University, Tianjin, China. [4]Institute of Urology, Peking University; National Urological Cancer Center; Beijing, China. [5]Department of Urology, People's Hospital of Rizhao, Rizhao, Shandong, China.

[*]Corresponding author. Tel: +86 022 88328607; E-mail: quancahngyi@tmu.edu.cn; ORCID: 0000-0002-3511-5604

[**]Corresponding author. Tel: +86 022 88329296; E-mail: zhiqun_shang@tmu.edu.cn; ORCID: 0000-0001-8263-707X

[***]Corresponding author. Tel: +86 022 88329296; E-mail: niuyuanjie9317@163.com; ORCID: 0000-0002-9266-727X

Shimiao Zhu, Zhao Yang, Zheng Zhang and Hongli Zhang contributed equally to this work.

Zhiqun Shang PhD&MD
Professor of Tianjin Institute of Urology
The Second Hospital of Tianjin Medical University
TEL:18722292731

快捷回复全部

Re:Authorship Confirmation

ToMe

X Xuanrong 2023-03-12 13:52
收件人：我 详情

Dear Prof. Shang,

I agree with this authorship.

Shimiao Zhu

发件人："尚芝群" <zhiqun_shang@tmu.edu.cn>
发送日期：2023-03-12 10:35:59
收件人：Xuanrong <chan@tmu.edu.cn>
主题：Authorship Confirmation

隐藏引用内容

Dear Dr. Chen,

As determined after discussion, the authorship has been updated as listed in the figure at bottom. Before publication, agreements from all co-authors should be suggestion.
Thank you!

Shimiao Zhu[1], Zhao Yang[1], Zheng Zhang[1], Hongli Zhang[1], Songyang Li[1], Tao Wu[2], Xuanrong Chen[1], Jianing Guo[3], Aixiang Wang[4], Hao Tian[1], Jianpeng Yu[1], Changwen Zhang[1], Lei Su[5], Zhiqun Shang[1,**], Changyi Quan[1,*], Yuanjie Niu[1,***]

[1]Department of Urology, Tianjin Institute of Urology, The Second Hospital of Tianjin Meidical University, Tianjin, 300211, China. [2]Department of Urology, Shenzhen Hospital, Southern Medical University, Shenzhen, China. [3]Department of Pathology, The Second Hospital of Tianjin Medical University, Tianjin, China. [4]Institute of Urology, Peking University; National Urological Cancer Center; Beijing, China. [5]Department of Urology, People's Hospital of Rizhao, Rizhao, Shandong, China.

[*]Corresponding author. Tel: +86 022 88328607; E-mail: quancahngyi@tmu.edu.cn; ORCID: 0000-0002-3511-5604

[**]Corresponding author. Tel: +86 022 88329296; E-mail: zhiqun_shang@tmu.edu.cn; ORCID: 0000-0001-8263-707X

[***]Corresponding author. Tel: +86 022 88329296; E-mail: niuyuanjie9317@163.com; ORCID: 0000-0002-9266-727X

Shimiao Zhu, Zhao Yang, Zheng Zhang and Hongli Zhang contributed equally to this work.

Zhiqun Shang PhD&MD
Professor of Tianjin Institute of Urology
The Second Hospital of Tianjin Medical University
TEL:18722292731

Re:Authorship Confirmation

ToMe

J Jianing 2023-03-12 10:58
收件人：我 详情

Dear Prof. Shang,

I agree with this authorship.

Jianing Guo

发件人："尚芝群" <zhiqun_shang@tmu.edu.cn>
发送日期：2023-03-12 10:35:59
收件人：Jianing <Guojianing2005@163.com>
主题：Authorship Confirmation

隐藏引用内容

Dear Dr. Guo,

As determined after discussion, the authorship has been updated as listed in the figure at bottom. Before publication, agreements from all co-authors should be suggestion.
Thank you!

Shimiao Zhu[1], Zhao Yang[1], Zheng Zhang[1], Hongli Zhang[1], Songyang Li[1], Tao Wu[2], Xuanrong Chen[1], Jianing Guo[3], Aixiang Wang[4], Hao Tian[1], Jianpeng Yu[1], Changwen Zhang[1], Lei Su[5], Zhiqun Shang[1,**] , Changyi Quan[1,*], Yuanjie Niu[1,***]

[1]Department of Urology, Tianjin Institute of Urology, The Second Hospital of Tianjin Meidical University, Tianjin, 300211, China. [2]Department of Urology, Shenzhen Hospital, Southern Medical University, Shenzhen, China. [3]Department of Pathology, The Second Hospital of Tianjin Medical University, Tianjin, China. [4]Institute of Urology, Peking University; National Urological Cancer Center; Beijing, China. [5]Department of Urology, People's Hospital of Rizhao, Rizhao, Shandong, China.

[*]Corresponding author. Tel: +86 022 88328607; E-mail: quancahngyi@tmu.edu.cn; ORCID: 0000-0002-3511-5604

[**]Corresponding author. Tel: +86 022 88329296; E-mail: zhiqun_shang@tmu.edu.cn; ORCID: 0000-0001-8263-707X

[***]Corresponding author. Tel: +86 022 88329296; E-mail: niuyuanjie9317@163.com; ORCID: 0000-0002-9266-727X

Shimiao Zhu, Zhao Yang, Zheng Zhang and Hongli Zhang contributed equally to this work.

Zhiqun Shang PhD&MD
Professor of Tianjin Institute of Urology
The Second Hospital of Tianjin Medical University
TEL:18722292731

Re:Authorship Confirmation

ToMe

A Aixiang 2023-03-12 16:35
收件人：我 详情

Dear Prof. Shang,

I agree with this authorship.

Aixiang Wang

发件人："尚芝群" <zhiqun_shang@tmu.edu.cn>
发送日期：2023-03-12 10:35:59
收件人：Aixiang <wax20030826@126.com>
主题：Authorship Confirmation

隐藏引用内容

Dear Dr. Wang,

As determined after discussion, the authorship has been updated as listed in the figure at bottom. Before publication, agreements from all co-authors should be suggestion.
Thank you!

Shimiao Zhu[1], Zhao Yang[1], Zheng Zhang[1], Hongli Zhang[1], Songyang Li[1], Tao Wu[2], Xuanrong Chen[1], Jianing Guo[3], Aixiang Wang[4], Hao Tian[1], Jianpeng Yu[1], Changwen Zhang[1], Lei Su[5], Zhiqun Shang[1,**] , Changyi Quan[1,*], Yuanjie Niu[1,***]

[1]Department of Urology, Tianjin Institute of Urology, The Second Hospital of Tianjin Meidical University, Tianjin, 300211, China. [2]Department of Urology, Shenzhen Hospital, Southern Medical University, Shenzhen, China. [3]Department of Pathology, The Second Hospital of Tianjin Medical University, Tianjin, China. [4]Institute of Urology, Peking University; National Urological Cancer Center; Beijing, China. [5]Department of Urology, People's Hospital of Rizhao, Rizhao, Shandong, China.

[*]Corresponding author. Tel: +86 022 88328607; E-mail: quancahngyi@tmu.edu.cn; ORCID: 0000-0002-3511-5604

[**]Corresponding author. Tel: +86 022 88329296; E-mail: zhiqun_shang@tmu.edu.cn; ORCID: 0000-0001-8263-707X

[***]Corresponding author. Tel: +86 022 88329296; E-mail: niuyuanjie9317@163.com; ORCID: 0000-0002-9266-727X

Shimiao Zhu, Zhao Yang, Zheng Zhang and Hongli Zhang contributed equally to this work.

Zhiqun Shang PhD&MD
Professor of Tianjin Institute of Urology
The Second Hospital of Tianjin Medical University
TEL:18722292731

Re:Authorship Confirmation

ToMe

T 田昊 2023-03-12 11:59
收件人：我 详情

Dear Prof. Shang,

I agree with this authorship.

Hao Tian

发件人："尚芝群"
<zhiqun_shang@tmu.edu.cn>发送日期：
2023-03-12 10:35:59
收件人：田昊 <yjs_tianhao@tmu.edu.cn>
主题：Authorship Confirmation
隐藏引用内容

Dear Dr. Tian,

As determined after discussion, the authorship has been updated as listed in the figure at bottom. Before publication, agreements from all co-authors should be suggestion.
Thank you!

Shimiao Zhu[1], Zhao Yang[1], Zheng Zhang[1], Hongli Zhang[1], Songyang Li[1], Tao Wu[2], Xuanrong Chen[1], Jianing Guo[3], Aixiang Wang[4], Hao Tian[1], Jianpeng Yu[1], Changwen Zhang[1], Lei Su[5], Zhiqun Shang[1,**], Changyi Quan[1,*], Yuanjie Niu[1,***]

[1]Department of Urology, Tianjin Institute of Urology, The Second Hospital of Tianjin Meidical University, Tianjin, 300211, China. [2]Department of Urology, Shenzhen Hospital, Southern Medical University, Shenzhen, China. [3]Department of Pathology, The Second Hospital of Tianjin Medical University, Tianjin, China. [4]Institute of Urology, Peking University; National Urological Cancer Center; Beijing, China. [5]Department of Urology, People's Hospital of Rizhao, Rizhao, Shandong, China.

[*]Corresponding author. Tel: +86 022 88328607; E-mail: quancahngyi@tmu.edu.cn; ORCID: 0000-0002-3511-5604

[**]Corresponding author. Tel: +86 022 88329296; E-mail: zhiqun_shang@tmu.edu.cn; ORCID: 0000-0001-8263-707X

[***]Corresponding author. Tel: +86 022 88329296; E-mail: niuyuanjie9317@163.com; ORCID: 0000-0002-9266-727X

Shimiao Zhu, Zhao Yang, Zheng Zhang and Hongli Zhang contributed equally to this work.

Zhiqun Shang PhD&MD
Professor of Tianjin Institute of Urology
The Second Hospital of Tianjin Medical University
TEL:18722292731

Re:Authorship Confirmation

ToMe

J Jianpeng 2023-03-12 12:01
收件人：我 详情

Dear Prof. Shang,

I agree with this authorship.

Jianpeng Yu

发件人："尚芝群" <zhiqun_shang@tmu.edu.cn>
发送日期：2023-03-12 10:35:59
收件人：Jianpeng <yujianpeng@tmu.edu.cn>
主题：Authorship Confirmation

隐藏引用内容

Dear Dr. Yu,

As determined after discussion, the authorship has been updated as listed in the figure at bottom. Before publication, agreements from all co-authors should be suggestion.
Thank you!

Shimiao Zhu[1], Zhao Yang[1], Zheng Zhang[1], Hongli Zhang[1], Songyang Li[1], Tao Wu[2], Xuanrong Chen[1], Jianing Guo[3], Aixiang Wang[4], Hao Tian[1], Jianpeng Yu[1], Changwen Zhang[1], Lei Su[5], Zhiqun Shang[1,**], Changyi Quan[1,*], Yuanjie Niu[1,***]

[1]Department of Urology, Tianjin Institute of Urology, The Second Hospital of Tianjin Meidical University, Tianjin, 300211, China. [2]Department of Urology, Shenzhen Hospital, Southern Medical University, Shenzhen, China. [3]Department of Pathology, The Second Hospital of Tianjin Medical University, Tianjin, China. [4]Institute of Urology, Peking University; National Urological Cancer Center; Beijing, China. [5]Department of Urology, People's Hospital of Rizhao, Rizhao, Shandong, China.

[*]Corresponding author. Tel: +86 022 88328607; E-mail: quancahngyi@tmu.edu.cn; ORCID: 0000-0002-3511-5604

[**]Corresponding author. Tel: +86 022 88329296; E-mail: zhiqun_shang@tmu.edu.cn; ORCID: 0000-0001-8263-707X

[***]Corresponding author. Tel: +86 022 88329296; E-mail: niuyuanjie9317@163.com; ORCID: 0000-0002-9266-727X

Shimiao Zhu, Zhao Yang, Zheng Zhang and Hongli Zhang contributed equally to this work.

Zhiqun Shang PhD&MD
Professor of Tianjin Institute of Urology
The Second Hospital of Tianjin Medical University
TEL:18722292731

快捷回复全部

zhiqun_shang@tmu.edu.cn 设置 下载 帮助与反馈 English 退出登录 回到旧版

收件箱 Authorship ... 系统退信 Fw:CDDIS-2... 无主题 Authorship ... Track the sta... Re:Authorsh...

邮箱 12 日历 通讯录 消息 云文档

写邮件

搜索邮件

- 收件箱
- 未读邮件 28
- 发件箱
- 红旗邮件
- 任务邮件
- 稍后处理
- 草稿箱 11
- 垃圾邮件
- 已删除
- 广告邮件
- 订阅邮件

新建文件夹

自定义专属文件夹，高效管理各类邮件

新建文件夹

标签

返回 回复 回复全部 转发 删除 标记为 移动 举报 分享 更多

Re:Authorship Confirmation

ToMe

Changwen 2023-03-12 17:03
收件人：我 详情

Dear Prof. Shang,

I agree with this authorship.

Changwen Zhang

发件人："尚芝群" <zhiqun_shang@tmu.edu.cn>
发送日期：2023-03-12 10:35:59
收件人：Changwen <zhangchangwen05@tmu.edu.cn>
主题：Authorship Confirmation

隐藏引用内容

Dear Dr. Zhang,

As determined after discussion, the authorship has been updated as listed in the figure at bottom. Before publication, agreements from all co-authors should be suggestion.
Thank you!

Shimiao Zhu[1], Zhao Yang[1], Zheng Zhang[1], Hongli Zhang[1], Songyang Li[1], Tao Wu[2], Xuanrong Chen[1], Jianing Guo[3], Aixiang Wang[4], Hao Tian[1], Jianpeng Yu[1], Changwen Zhang[1], Lei Su[5], Zhiqun Shang[1,**], Changyi Quan[1,*], Yuanjie Niu[1,***]

[1]Department of Urology, Tianjin Institute of Urology, The Second Hospital of Tianjin Meidical University, Tianjin, 300211, China. [2]Department of Urology, Shenzhen Hospital, Southern Medical University, Shenzhen, China. [3]Department of Pathology, The Second Hospital of Tianjin Medical University, Tianjin, China. [4]Institute of Urology, Peking University; National Urological Cancer Center; Beijing, China. [5]Department of Urology, People's Hospital of Rizhao, Rizhao, Shandong, China.

[*]Corresponding author. Tel: +86 022 88328607; E-mail: quancahngyi@tmu.edu.cn; ORCID: 0000-0002-3511-5604

[**]Corresponding author. Tel: +86 022 88329296; E-mail: zhiqun_shang@tmu.edu.cn; ORCID: 0000-0001-8263-707X

[***]Corresponding author. Tel: +86 022 88329296; E-mail: niuyuanjie9317@163.com; ORCID: 0000-0002-9266-727X

Shimiao Zhu, Zhao Yang, Zheng Zhang and Hongli Zhang contributed equally to this work.

Zhiqun Shang PhD&MD
Professor of Tianjin Institute of Urology
The Second Hospital of Tianjin Medical University
TEL:18722292731

快捷回复全部

Re:Authorship Confirmation

ToMe

L Lei 2023-03-12 17:51
收件人：我 详情

Dear Prof. Shang,

I agree this authorship.

Lei Su

发件人："尚芝群" <zhiqun_shang@tmu.edu.cn>
发送日期：2023-03-12 10:35:59
收件人：Lei <345020147@qq.com>
主题：Authorship Confirmation

隐藏引用内容

Dear Dr. Su,

As determined after discussion, the authorship has been updated as listed in the figure at bottom. Before publication, agreements from all co-authors should be suggestion.
Thank you!

Shimiao Zhu[1], Zhao Yang[1], Zheng Zhang[1], Hongli Zhang[1], Songyang Li[1], Tao Wu[2], Xuanrong Chen[1], Jianing Guo[3], Aixiang Wang[4], Hao Tian[1], Jianpeng Yu[1], Changwen Zhang[1], Lei Su[5], Zhiqun Shang[1,**], Changyi Quan[1,*], Yuanjie Niu[1,***]

[1]Department of Urology, Tianjin Institute of Urology, The Second Hospital of Tianjin Meidical University, Tianjin, 300211, China. [2]Department of Urology, Shenzhen Hospital, Southern Medical University, Shenzhen, China. [3]Department of Pathology, The Second Hospital of Tianjin Medical University, Tianjin, China. [4]Institute of Urology, Peking University; National Urological Cancer Center; Beijing, China. [5]Department of Urology, People's Hospital of Rizhao, Rizhao, Shandong, China.

[*]Corresponding author. Tel: +86 022 88328607; E-mail: quancahngyi@tmu.edu.cn; ORCID: 0000-0002-3511-5604

[**]Corresponding author. Tel: +86 022 88329296; E-mail: zhiqun_shang@tmu.edu.cn; ORCID: 0000-0001-8263-707X

[***]Corresponding author. Tel: +86 022 88329296; E-mail: niuyuanjie9317@163.com; ORCID: 0000-0002-9266-727X

Shimiao Zhu, Zhao Yang, Zheng Zhang and Hongli Zhang contributed equally to this work.

Zhiqun Shang PhD&MD
Professor of Tianjin Institute of Urology
The Second Hospital of Tianjin Medical University
TEL:18722292731

Re:Authorship Confirmation

ToMe

Q Quan 2023-03-12 11:02
收件人：我 详情

Dear Prof. Shang,

I agree this authorship.

Changyi Quan

发件人："尚芝群" <zhiqun_shang@tmu.edu.cn>
发送日期：2023-03-12 10:35:59
收件人：Quan <quanchangyi@tmu.edu.cn>主
题：Authorship Confirmation

隐藏引用内容

Dear Dr. Quan

As determined after discussion, the authorship has been updated as listed in the figure at bottom. Before publication, agreements from all co-authors should be suggestion.
Thank you!

Shimiao Zhu[1], Zhao Yang[1], Zheng Zhang[1], Hongli Zhang[1], Songyang Li[1], Tao Wu[2], Xuanrong Chen[1], Jianing Guo[3], Aixiang Wang[4], Hao Tian[1], Jianpeng Yu[1], Changwen Zhang[1], Lei Su[5], Zhiqun Shang[1,**], Changyi Quan[1,*], Yuanjie Niu[1,***]

[1]Department of Urology, Tianjin Institute of Urology, The Second Hospital of Tianjin Meidical University, Tianjin, 300211, China. [2]Department of Urology, Shenzhen Hospital, Southern Medical University, Shenzhen, China. [3]Department of Pathology, The Second Hospital of Tianjin Medical University, Tianjin, China. [4]Institute of Urology, Peking University; National Urological Cancer Center; Beijing, China. [5]Department of Urology, People's Hospital of Rizhao, Rizhao, Shandong, China.

*Corresponding author. Tel: +86 022 88328607; E-mail: quancahngyi@tmu.edu.cn; ORCID: 0000-0002-3511-5604

**Corresponding author. Tel: +86 022 88329296; E-mail: zhiqun_shang@tmu.edu.cn; ORCID: 0000-0001-8263-707X

***Corresponding author. Tel: +86 022 88329296; E-mail: niuyuanjie9317@163.com; ORCID: 0000-0002-9266-727X

Shimiao Zhu, Zhao Yang, Zheng Zhang and Hongli Zhang contributed equally to this work.

Zhiqun Shang PhD&MD
Professor of Tianjin Institute of Urology
The Second Hospital of Tianjin Medical University
TEL:18722292731

zhiqun_shang@tmu.edu.cn 设置 下载 帮助与反馈 English 退出登录 回到旧版

收件箱 | Authorship ... | 系统退信 | Fw:CDDIS-2... | 无主题 | Authorship ... | Track the sta... | Re:Authorsh...

邮箱 12 日历 通讯录 消息 云文档

写邮件

搜索邮件

- 收件箱
- 未读邮件 28
- 发件箱
- 红旗邮件
- 任务邮件
- 稍后处理
- 草稿箱 11
- 垃圾邮件
- 已删除
- 广告邮件
- 订阅邮件

新建文件夹

自定义专属文件夹，高效管理各类邮件

新建文件夹

标签

返回 回复 回复全部 转发 删除 标记为 移动 举报 分享 更多

Re:Authorship Confirmation

ToMe

Y Yuanjie 2023-03-12 10:58
收件人：我 详情

Dear Prof. Shang,

I agree with this authorship.

Yuanjie Niu

发件人："尚芝群" <zhiqun_shang@tmu.edu.cn>
发送日期：2023-03-12 10:35:59
收件人：Yuanjie <niuyuanjie9317@163.com>
主题：Authorship Confirmation

隐藏引用内容

Dear Prof. Niu,

As determined after discussion, the authorship has been updated as listed in the figure at bottom. Before publication, agreements from all co-authors should be suggestion.
Thank you!

Shimiao Zhu[1], Zhao Yang[1], Zheng Zhang[1], Hongli Zhang[1], Songyang Li[1], Tao Wu[2], Xuanrong Chen[1], Jianing Guo[3], Aixiang Wang[4], Hao Tian[1], Jianpeng Yu[1], Changwen Zhang[1], Lei Su[5], Zhiqun Shang[1,**], Changyi Quan[1,*], Yuanjie Niu[1,***]

[1]Department of Urology, Tianjin Institute of Urology, The Second Hospital of Tianjin Meidical University, Tianjin, 300211, China. [2]Department of Urology, Shenzhen Hospital, Southern Medical University, Shenzhen, China. [3]Department of Pathology, The Second Hospital of Tianjin Medical University, Tianjin, China. [4]Institute of Urology, Peking University; National Urological Cancer Center; Beijing, China. [5]Department of Urology, People's Hospital of Rizhao, Rizhao, Shandong, China.

[*]Corresponding author. Tel: +86 022 88328607; E-mail: quancahngyi@tmu.edu.cn; ORCID: 0000-0002-3511-5604

[**]Corresponding author. Tel: +86 022 88329296; E-mail: zhiqun_shang@tmu.edu.cn; ORCID: 0000-0001-8263-707X

[***]Corresponding author. Tel: +86 022 88329296; E-mail: niuyuanjie9317@163.com; ORCID: 0000-0002-9266-727X

Shimiao Zhu, Zhao Yang, Zheng Zhang and Hongli Zhang contributed equally to this work.

Zhiqun Shang PhD&MD
Professor of Tianjin Institute of Urology
The Second Hospital of Tianjin Medical University
TEL:18722292731

快捷回复全部
